# Clinical Practice: Should we Radically Alter our Sedation of Critical Care Patients, Especially Given the COVID-19 Pandemics?

**DOI:** 10.2478/rjaic-2020-0018

**Published:** 2021-01-04

**Authors:** D Longrois, F Petitjeans, O Simonet, M de Kock, M Belliveau, C Pichot, Th Lieutaud, M Ghignone, L Quintin

**Affiliations:** 1 Départements d’Anesthésie-Réanimation, Université Paris-Diderot and Paris VII Sorbonne-Paris-Cité, Hôpital Bichat-Claude Bernard, Assistance Publique-Hôpitaux de Paris and UMR 5698, Paris, France; 2 Hôpital d’Instruction des Armées Desgenettes, Lyon, France; 3 Centre Hospitalier de Wallonie Picarde, Tournai, Belgium; 4 Hôpital de St Jerome, St Jérôme, Québec, Canada; 5 Hôpital Louis Pasteur, Dole, France; 6 Hôpital de Bourg en Bresse Bourg-en-Bresse France; 7 Centre de Recherche en Neurosciences (TIGER,UMR CRNS 5192-INSERM 1098), Lyon-Bron, France; 8 J.F. Kennedy Hospital North Campus, West Palm Beach, Florida, USA

**Keywords:** Critical care, sedation, general anaesthesia, conventional sedation, cooperative sedation, alpha-2 adrenergic agonists, alpha-2 agonists, clonidine, dexmedetomidine, guanfacine

## Abstract

The high number of patients infected with the SARS-CoV-2 virus requiring care for ARDS puts sedation in the critical care unit (CCU) to the edge. Depth of sedation has evolved over the last 40 years (no-sedation, deep sedation, daily emergence, minimal sedation, etc.). Most guidelines now recommend determining the depth of sedation and minimizing the use of benzodiazepines and opioids. The broader use of alpha-2 adrenergic agonists (‘alpha-2 agonists’) led to sedation regimens beginning at admission to the CCU that contrast with hypnotics+opioids (“conventional“ sedation), with major consequences for cognition, ventilation and circulatory performance. The same doses of alpha-2 agonists used for ‘cooperative’ sedation (ataraxia, analgognosia) elicit no respiratory depression but modify the autonomic nervous system (cardiac parasympathetic activation, attenuation of excessive cardiac and vasomotor sympathetic activity). Alpha-2 agonists should be selected only in patients who benefit from their effects (‘personalized’ indications, as opposed to a ‘one size fits all’ approach). Then, titration to effect is required, especially in the setting of systemic hypotension and/or hypovolemia. Since no general guidelines exist for the use of alpha-2 agonists for CCU sedation, our clinical experience is summarized for the benefit of physicians in clinical situations in which a recommendation might never exist (refractory delirium tremens; unstable, hypovolemic, hypotensive patients, etc.). Because the physiology of alpha-2 receptors and the pharmacology of alpha-2 agonists lead to personalized indications, some details are offered. Since interactions between conventional sedatives and alpha-2 agonists have received little attention, these interactions are addressed. Within the existing guidelines for CCU sedation, this article could facilitate the use of alpha-2 agonists as effective and safe sedation while awaiting large, multicentre trials and more evidence-based medicine.

## Introduction: A global shift in the depth of sedation and drug regimens for critical care sedation

The use of deep sedation, that is, *de facto*[1] general anaesthesia, in the critical care unit (CCU) generated concerns.[1] Accordingly, US guidelines for CCU sedation (conventional sedation, ‘analgo-sedation’, or light total intravenous anaesthesia [TIVA]: midazolam+sufentanil or propofol+remifentanil) have changed the emphasis from benzodiazepines and opioids use[2] towards analgo-sedation without opioids (‘opioid-free analgo-sedation’), with no/minimal benzodiazepines use. Indeed, high plasma concentrations of benzodiazepines and opioids are associated with CCU delirium.[3] The German societies of medicine have supported this trend[4] and suggested major changes.[5] Currently, the emphasis of sedation strategies is on prevention and treatment of CCU delirium. Thus, the implication is that the patient does *not* need to be ‘asleep and motionless’ but rather alert to *actively* participate to daily care.[4] The depth of sedation, from its initiation, is related to the duration of mechanical ventilation, hospital mortality and long-term outcomes.[6] Indeed, the pendulum swung back[7] from continuous sedation to interrupted sedation,[8] ‘no sedation’[9] and now to ‘cooperative sedation’, a new indication for old drugs. Alpha-2 adrenergic agonists (‘alpha-2 agonists’: clonidine, guanfacine) were used in the ’60–’80s as the second-generation sympatholytics and antihypertensive agents in the cardiology setting. They are currently used in the CCU as sedative agents (dexmedetomidine, clonidine) since they reduce the affective-motivational component of pain (indifference to pain, ‘analgognosia’)[10] and evoke indifference to the environment (‘ataraxia’)1After clonidine 300 mg p.o., volunteers switch easily from light sleep to wakefulness (‘fairly alert’) and back.[11] In the CCU, under dexmedetomidine: a) an intubated child played a game of little horses with his nurse (P Delaire, RN, personal communication); and b) an intubated patient reported ischemic chest pain, allowing for treatment.[12]. A landmark article[9] randomized patients under pressure support (PS) ventilation to ‘no sedation’ vs. sedation ‘as usual’ (‘no sedation’: morphine 2.5 to 5-mg bolus as needed, verbal reassurance as needed by an extra individual, and haloperidol 1–5 mg when delirium was observed, followed by a propofol infusion if the patient seemed uncomfortable; ‘as usual’: morphine 2.5 to 5 mg bolus as needed, propofol infusion to a Ramsay score of 3–4, and daily awakening). No sedation resulted in a greater need for reassurance, a higher incidence of delirium (no sedation: 20%; sedation: 7%), fewer ventilation days (p < 0.02), shorter CCU and hospital stays (p < 0.05), lower CCU and hospital mortality (non-significant: ns),[9] and no long-term psychological sequelae.[13] The same investigators randomized patients to light sedation in both groups (no sedation: RASS: -1.3/0.7 vs. light sedation: -2.3/-1.8) with survival unaffected.[14] By contrast, In agreement with,[6] deep sedation affects survival. This report[9] lays the groundwork for the introduction of ‘cooperative’ sedation, which is defined here as sedation achieved with alpha-2 agonists as *first-line* sedative agents.[15, 16, 17]

The German guidelines[4] distinguish sedation strategies for pregnant and breastfeeding women; end-of-life patients; patients with severe burns, multiple trauma, or intracranial hypertension; post-cardiac surgery patients; and patients on extracorporeal membrane oxygenation (ECMO) or in the prone position. The present manuscript is restricted to clinical situations (part II: alcohol withdrawal, refractory delirium tremens (DT), hypotensive/hypovolemic patients, multiple trauma and cardiac surgery patients), in which we[15,17] have gained expertise since 1980 in the US, Quebec, Belgium and France. Admittedly, this manuscript delineates the clinical reasoning based on pathophysiological/pharmacological lineaments in the *absence* of recommendations, evidence-based medicine or guidelines. Presumably, guidelines will never be issued in settings such as refractory DT, hypovolemic or frail patients. The core message is to use alpha-2 agonists as *first-line* sedatives,[15, 16, 17] avoiding further aggravation of a patient’s condition, such as exacerbated circulatory instability or CCU-acquired pathologies (emergence delirium, myoneuropathy, immuno-paralysis,[18] malnutrition, etc.). We will take advantage of this article to propose several detailed video classes on the use of alpha-2 agonists in specific situations.

This article includes the following formatting.

- Part I: a rationale for the use of alpha-2 agonists in CCU sedation. Given the paucity of evidence-based data, speculations are separated from the remainder of the manuscript: the notation used by physiologists[19, 20, 21] **(**[…..]**)** is used with a different font.
- Part II: the use of alpha-2 agonists is delineated in specific settings (alcohol withdrawal, refractory delirium tremens (DT), hypotensive/hypovolemic patients, multiple trauma and cardiac surgery patients). Basic elements are provided in inserts I and II for understanding the autonomic nervous system physiology, the pharmacology of alpha-2 agonists, and the consequences with respect to adequate volume replacement before administering alpha-2 agonists. Abridged tables translate guidelines[22] into personalized clinical reasoning in these settings.

## Part I: Rationale for the use of alpha-2 agonists in CCU sedation

Several arguments suggest using alpha-2 agonists for CCU sedation: pharmacology (opioid-sparing effects, analgesia, sedation mimicking physiological stage 2 sleep), pathophysiology (modified autonomic nervous system, spontaneous ventilation, noninvasive ventilation [NIV]) and outcome-related.[23, 24, 25, 26, 27, 28]

### I) Rationale for the use of alpha-2 agonists in the CCU

**Figure 1:**
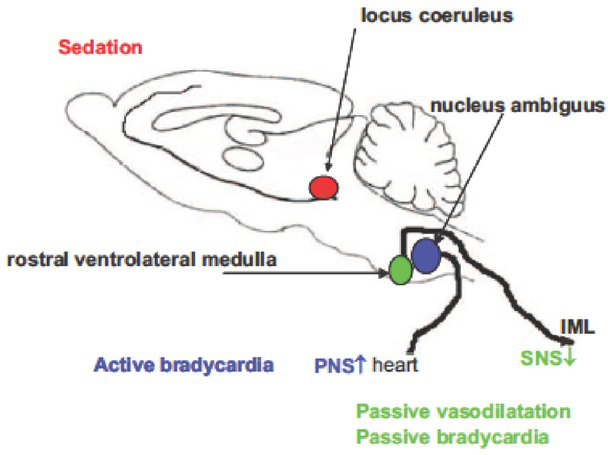
**Impact of alpha-2 agonists on the noradrenergic dorsal bundle, adrenergic ventral bundle and cardiac parasympathetic motoneurons**. Modified from Pichot, J Intens Care Med, 2012, 27 : 219–37.[15] a) Following depletion of the central and peripheral noradrenergic/adrenergic systems by reserpine, a bolus of a low-dose alpha-2 agonist generates hypertension (Kobinger 1967 quoted from (147)): hypertension is a consequence of an action on peripheral vascular alpha-1 receptors and extravascular alpha-2 receptors themselves (Figure 3), independent of the release of noradrenaline, that is, *independent* of the sympathetic nervous system. The observation of a peripheral effect of the alpha-2 agonist after depletion of sympathetic nerves (Kobinger, 1967) is replicated centrally: the anaesthetic-sparing effect of an alpha-2 agonist is observed after full destruction of catecholaminergic cell bodies (Figure 5 in (216)). Consequently, alpha-2 agonists cannot evoke sedation via pre-synaptic autoreceptors located on catecholaminergic cell bodies. The claim that alpha-2 agonists act through pre-synaptic alpha-2 autoreceptors located on noradrenergic locus coeruleus cell bodies[46,217] is misleading. A nuanced discussion appears in (50, 218). b) The alpha-2 agonists inhibit the adrenergic and glutamatergic neurons[219] located in the rostral ventrolateral medulla (pre-sympathetic neurons in the RVLM ‘vasomotor centre’), decreasing the set point of the vascular sympathetic baroreflex without diminishing the neurons’ reactivity to decreased systemic pressure (220). The pre-sympathetic adrenergic and glutamatergic neurons project from the lower brain stem to the spinal intermediolateral cell column, from which sympathetic pre-ganglionic neurons originate. Alpha-2 agonists inhibit RVLM neurons, reduce cardiac sympathetic activity (reduced noradrenaline release leading to slow ‘*passive*’ bradycardia in the presence of a slow intrasynaptic NA reuptake) and reduce vascular sympathetic activity (slow ‘*passive’* vasodilation). c) Alpha-2 agonists stimulate alpha-2 receptors located on or near cardiac parasympathetic neurons (‘cardiac vagal motoneurons’) in the nucleus ambiguous external formation (dorsal and medial to the vasomotor centre). In a healthy, supine, resting volunteer, a microdose of vasopressor administered as a bolus (e.g., phenylephrine 10 to 30 *m*g) evokes minimal increase in systemic pressure next to the resting, baseline, pressure, stimulates the cardiac parasympathetic neurons and evokes fast ‘active’ bradycardia,[221] owing to *active* acetylcholine release and rapid intrasynaptic breakdown. This process increases the slope of the pressure-RR interval relationship[222] (slope of the cardiac baroreflex ‘reactivity’).This rapid bradycardia evokes stabilization of the blood pressure within one beat. [223] in the supine resting healthy volunteer. The circulatory properties of the alpha-2 agonists are explained by: a) increased active cardiac parasympathetic reactivity, and b) normalized cardiac and vascular sympathetic activity. In the CCU patient, this process occurs in the setting of high baseline sympathetic activity and suppressed baseline parasympathetic activity. In addition, full normalization of increased sympathetic activity is not necessarily achieved, according to the dose of the alpha-2 agonist and the amplitude of the sympathetic activation. In contrast, in the healthy, supine volunteer, the baseline low normal sympathetic activity is deactivated to residual activity, while the cardiac parasympathetic activity is activated.

A) **Cardio-pulmonary interactions**
  An opioid-free analgo-sedation strategy combining non-opioid analgesics and alpha-2 agonists does not cause respiratory depression. This absence of respiratory depression combined with indifference to the environment (‘ataraxia’) has a major impact, allowing one to use *continuous* NIV for an extended period.[29, 30, 31, 32, 33]2In ARDS patients (PaO2/FiO2 [P/F]: face mask: 144; helmet: 118; Acute Physiology And Chronic Health Evaluation: APACHE=26), helmet NIV rather than mask NIV leads to a shorter duration of ventilation and lower mortality [34]. This finding might be related to a higher positive end-expiratory pressure (PEEP) level and lower pressure support [35], i.e., “inverted settings” [36]. When possible, such a strategy circumvents tracheal intubation (‘intubation’) or reintubation after tracheal extubation3Tolerance to NIV is substantially increased under sedation with alpha-2 agonists.[37] Accordingly, the duration of invasive ventilation[37] is reduced by delaying intubation or allowing for early extubation.[37] (‘extubation’), which could limit the use of controlled invasive mechanical ventilation (CMV) and the administration of neuromuscular blockers (NMBs). In turn, spontaneous breathing evokes improved cardio-ventilatory interactions[21] and lowers vasopressor requirements[38, 39, 40] and possibly volume requirements. During the COVID-19 pandemics, many patients required high-flow oxygen therapy for days/weeks. As alpha-2 agonists do not depress the respiratory drive,[41] they were our first-line drugs with drug associations delineated below.
B) **Alpha-2 agonists**: anti-hypertensive vs. sedative agents? In the 1960s–1970s, the regulatory approval of alpha-2 agonists (clonidine, guanfacine, etc.) and imidazolines (rimelidine, etc.) as anti-hypertensive agents led to the equating of alpha-2 agonists with centrally acting anti-hypertensive agents, attenuating back towards baseline any possible excessive sympathetic activity targeted to the heart and vessels (‘cardiac and vasomotor sympathetic activity’ normalized to baseline activity). In the 1990–2000s, the regulatory approval of dexmedetomidine as a sedative for CCU sedation led to the equating of alpha-2 agonists with anaesthetic adjuncts[42] or sedative[43] agents. Both views are *misleading*: clonidine was synthetized as a nasal decongestant and then administered to a human ‘volunteer’, unexpectedly resulting in prolonged sleep, bradycardia and hypotension following nasal administration of the equivalent of 20 pills of clonidine.[44] Deducing sedative properties from circulatory properties[45] or vice-versa[46] is irrelevant: the doses of alpha-2 agonists necessary to achieve sedation are *identical* to the doses that evoke bradycardia or hypotension. In healthy volunteers, heart rate (HR) decreases (-10 to -20%) and normalization of blood pressure after the initial hypotensive phase are surrogate markers of sedation (40 < Bispectral Index (BIS) <60; more than 80% probability of loss of responsiveness).[47,48] The circulatory and cognitive/analgesic effects of alpha-2 agonists are anatomically and functionally[49] unrelated[50] part II, insert I). Indeed, the only common link is pharmacological, i.e., the alpha-2 adrenergic *receptor* itself. Since this alpha-2 adrenergic receptor is widely distributed[51,52] in the central (brain and spine) and autonomic (central and peripheral) nervous systems (Figure 1), multiple effects occur, including autonomic and cognitive effects. Indeed, a brief inventory (I, D) suggests that alpha-2 agonists present miraculous pharmacodynamics (‘*wonder drugs*’[53]). More simply, because these agents act via the sympathetic and parasympathetic systems and dorsal noradrenergic bundle (part II, insert I), they have numerous targets, via widely distributed central alpha-2 receptors[51,52] and numerous peripheral sites of action. The multiple effects explain their wide-ranging indications (hypertension, attention-deficit/hyperactivity, Tourette’s syndrome, rapid opioid detoxification, etc.), which are restricted here to CCU implications.[15,17,21,54]

**Figure 2:**
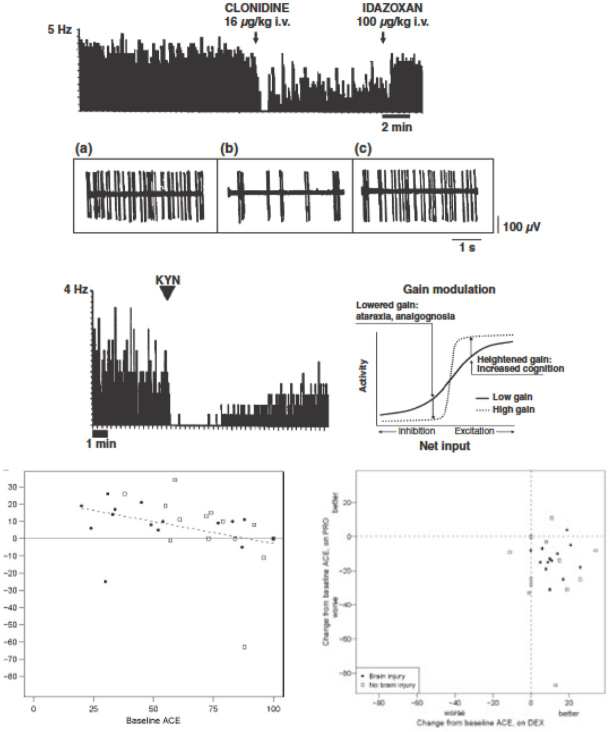
**Putative mechanism for cooperative sedation and minimization of CCU delirium following alpha-2 agonists**. *Top and upper middle*: In paralyzed rats emerging from halothane anaesthesia, during recording of locus coeruleus neurons following administration of clonidine, the slow baseline (‘tonic’) electrical activity observable between volleys of action potentials (‘phasic’ activity, middle inset a) is suppressed while leaving intact the phasic activity (middle inset b). Reversal of the effect of clonidine is achieved by the alpha-2 antagonist idazoxan (middle inset c). *Lower middle left*: a) A glutamate antagonist, kynurenic acid (KYN), micro-injected next to the locus coeruleus neuron, suppresses the remaining phasic electrical activity observed in middle inset b. Therefore, at least two components, alpha-2 mediated vs. glutamate mediated, are involved in the cooperative sedation evoked by the alpha-2 agonist. Modified from Saunier, Anesthesiology, 1993, 79, 1072–82.[224] *Lower middle right*: Putative functioning of the dorsal noradrenergic system following administration of an alpha-2 agonist. In a monkey presenting with hyperactivity, lack of attention, and poor discrimination between signal and detection tasks, a micro-injection of clonidine within the locus coeruleus reduces errors. This effect occurs possibly by lowering the tonic activity and increasing the phasic activity. Thus, alpha-2 agonists reinforce the filtering of the locus coeruleus toward a 0-1 functioning, that is, activation vs. inhibition. The neuron shifts towards a 0-1 functioning. An increase in gain (dotted lines) increases the activity of an LC neuron, which receives an excitatory stimulus (increased cognitive activity). The same increase in gain lowers the activity of the same neuron when it receives an inhibitory stimulus (indifference to environment: ataraxia). This schema accounts for the cooperative sedation observed in the CCU when a patient is left undisturbed and fully rousable upon stimulation. Modified from Servan-Schreiber, Science, 1990, 249: 892–5.[225] Aston-Jones, J Comp Neurol, 2005, 493: 99–110[226] and Pichot, Ann Fr Anesth Rean, 2012, 31: 876–96.[17] *Bottom left*: Following administration of dexmedetomidine, the cognitive function (adapted cognitive examination: ACE) increases more in patients with a worse initial condition, that is, lower baseline ACE, compared to propofol sedation (not shown). *Bottom right*: Changes in cognitive examination during propofol (ordinate) vs. dexmedetomidine (abscissae) from relative baseline in each of the 30 patients completing the study (black dot: brain injury; open dot: no brain injury). Note the few cognitive deteriorations following dexmedetomidine and few cognitive improvements following propofol sedation. Dexmedetomidine improves cognition more than propofol (p < 0.001). Modified from Mirski, *Intens Care Med*, 2010, 36, 1505–13.[75] Working memory is improved when an alpha-2 agonist, guanfacine is administered following mild traumatic brain injury.[227]

C) **Perspective**: normalized sympathetic activity vs. sedation The autonomic nervous system is a *coordinating* system, acting during ‘fight or flight’ responses,[55] ‘freezing behaviour’, diving reflex, exercise, sleep and so on. The sympathetic system coordinates circulation and ventilation via alpha- and beta-receptors.[56] However, in the CCU, a patient cannot initiate either a fight or flight response because: a) sympathetic hyperactivity lasts for days or weeks, at variance with exercise (except ultralong trails in fit athletes[57]); and b) cardiac parasympathetic (‘cardiac vagal’) activity is suppressed, in contrast to observations in ultralong trails.[57] Then, *after* stabilization of the acute cardio-ventilatory distress observed upon admission to the CCU, the sympathetic nervous system is not adaptive any more but *mal*-adaptive[58,59]^4^: at variance with rest and recovery of the healthy volunteer, and beyond the period preceding and following admission, adaptation in the CCU is of minimal value. Worse, coordination is lost. In the CCU, after days or weeks, the end result is an overflow of noradrenaline and an exhausted individual. This outcome has also been observed in very different settings (‘voodoo death’,[65] sudden death observed in the setting of burn-out: *karoshi*).
  The present hypothesis holds the following in the CCU:
  a) Beta blockers[66,67] and/or alpha-2 agonists attenuate excessive sympathetic hyperactivity. This hyperactivity is normalized pharmacologically toward baseline activity. (sympathetic de-activation, suppressed overflow). b) Increased sympathetic activity could be a factor limiting survival when increasing APACHE score[27] or age[68] is considered.
D) **Pharmacology**:
  The effects of alpha-2 agonists are multiple:
  1. Minimized stage 2 sleep fragmentation,[69, 70, 71] increased or decreased rapid-eye-movement (REM) sleep as a function of dose,[69,72] indifference to the environment (‘ataraxia’, ‘rousability’[43] or ‘cooperative’ sedation[48]), minimized emergence delirium,[73,74] improved cognition[75] (Figure 2), analgesia,[76,77] indifference to pain or the absence of awareness of pain (‘analgognosia’),[10] reduced tremor.[78,79] A patient’s comfort and cooperation in the CCU depend on these properties.
  2. No depression of the respiratory generator,[41,80] as opposed to observations with benzodiazepines, propofol and opioid analgesics, reduced hyperventilation5Of interest in the setting of weaning under pressure support ventilation to lower ventilator-to-patient dys-synchrony.[81][82] caused by pain or CCU-evoked distress, and a reduced duration of mechanical ventilation[37,83] and CCU stay.[84]
  3. Improved expiratory mechanics in the setting of asthma (increased peak expiratory flow rate and forced expiratory volume) [85]6Relevant in the setting of early ARDS (an intrinsic PEEP as high as 7 cm H20 is observed[86]) or severe bronchospasm/status asthmaticus.[87,33,32].
  4. Decreased sensitivity to hypercapnia [88], as observed during slow-wave sleep, and improved tolerance to permissive hypercapnia [81, 89]7Allowing one to set up a reduced tidal volume in the setting of acute respiratory distress syndrome (ARDS) in conjunction with fever control.[90].
  5. Decreased VO_2_ under heightened [91, 92] and baseline [93] conditions and decreased baseline temperature [88] leading to lowered temperature in the CCU [94], thus improving fever control [90].
  6. Reduced intrapulmonary shunting [95, 96].
  7. Reduced pulmonary hypertension [97, 98, 99] evoked by attenuated sympathetic hyperactivity [100].
  8. Decreased sinus node activity (bradycardia), slowed atrio-ventricular conduction (increased P-R interval [101]), and a reduced incidence of ventricular arrhythmias following cardiac surgery [102].
  9. In the setting of congestive heart failure: increased left ventricular (LV) diastolic [103] and systolic [104, 105, 106, 107] performance [104, 105, 106, 107]; experimentally: reduced arterial stiffness [108] and LV impedance [109].
  10. Improved coronary circulation [110] in the setting of myocardial infarction [111] or coronary surgery [112].
  11. Reduced cardiac filling pressure [104, 105] with effects depending on the state of the patient (improvement of cardiogenic pulmonary oedema [113] vs. aggravated hypotension when hypovolemia is present (part II, insert II).
  12. Vasodilation of skin vessels with increased toe temperature [49]; reduced vasopressor requirements in the setting of sepsis [25] and septic shock [38, 40, 114]. Presumably, from a circulatory point of view, by normalizing sympathetic hyperactivity, alpha-2 agonists re-couple the ventricle, the arterial circulation and the microcirculation.
  13. Increased diuresis in the setting of ascites,[115, 116, 117] cardiac failure[107] and critical care.[118]
  14. Improved renal function.[119,120]
  15. Reduced intra-abdominal pressure;[121] improved gastrointestinal motility.[122]
  16. Improved glucose control and reduced insulin requirements in type 2 diabetic patients undergoing surgery,[123] at variance with increased glycemia in healthy resting volunteers.
  17. Decreased procalcitonin[39] and pro-inflammatory interleukin-6[124, 125, 126] and increased anti-inflammatory interleukin-10.[125,127]
  In summary, alpha-2 agonists are associated with: a) sedation without cognitive impairment[75] (Figure 2);
  increased parasympathetic activity and decreased sympathetic activity. This occurs in contrast to the reduced parasympathetic activity and increased sympathetic activity generated by surgical or medical illness; and
  the absence of respiratory depression with possibly improved obstructive expiratory disease.[31, 32, 33,85] Despite such favourable pharmacodynamics, given the arterial hypotension, bradycardia or conduction block associated with the administration of alpha-2 agonists, alpha-2 agonists cannot be prescribed to all CCU patients. *Our view contrasts with the indications proposed by the US and European regulatory agencies*. We contend that only *personalized* prescriptions can be administered after: a) a full work-up (history, physical examination, electrocardiogram, echocardiography, CT scan, etc., if appropriate); and b) consideration of the benefits and risks for *each* patient. Cautious niche prescriptions do not preclude prescriptions to most CCU patients, but we oppose generalized routine prescriptions (‘one size fits all’).

F) **Outcomes**
  First, under conventional sedation, when a nurse is in charge of the moment-to-moment adaptation of sedation, the incidence of ventilation-associated pneumonia and the duration of mechanical ventilation are significantly reduced.[128] However, CCU or hospital mortality is reduced non-significantly.[128] Whether a similar benefit occurs with alpha-2 agonists is unknown. Second, whether attenuated sympathetic hyperactivity allows for recovery from the initial insult, minimizes CCU-acquired disease and improves outcome requires stronger evidence than available.[23, 24, 25, 26, 27, 28,68]

### II) Consequences of alpha-2 agonist use in the CCU: concerted nurse-physician changes

Since the patient is not any further under general anaesthesia, the objectives for a motionless CCU patient are to minimize pain, anxiety and autonomic hyperactivity, early transition from controlled to spontaneous ventilation, and facilitate full patient cooperation. This *Copernican* revolution in the CCU requires a *concerted* effort among nurses, physiotherapists and physicians. In contrast to previous practices,[1] this strategy requires changes with respect to the following:

1. Iterative evaluations of sedation (Ramsay scale, Richmond Agitation and Sedation Scale [RASS]), pain (Behavioural Pain Scale [BPS],[129] Visual Analogue Scale) and delirium (CAM-ICU)[130]; and
2. Adaptation of nurses, physiotherapists and physicians to continuous cooperative sedation in an alert, spontaneously breathing patient, with active maintenance of day-night cycle, patient reassurance and orientation, and early physiotherapy.

### III) Recommended doses

**Figure 3:**
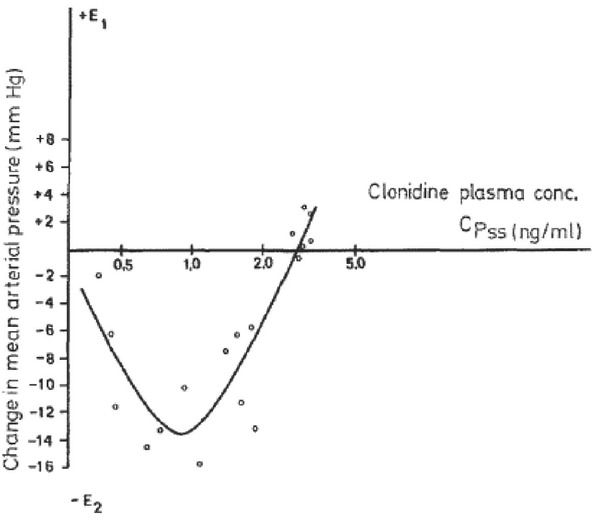
**Effect of increasing clonidine plasma concentrations on systemic blood pressure**. The lowest blood pressure is observed when the plasma clonidine concentration is 0.65±0.07 ng.mL-1. The effective pressor concentration is observed when the concentration is: 1.28±0.16 ng. mL-1. Modified from Frisk-Holmberg.[211] Therapeutic plasma concentrations for dexmedetomidine are 0.4-1.2 ng.mL-1.[228] Usual doses of clonidine 300 μg p.o. as administered in the cardiology setting achieve within 1–3 h (48) a large decrease in systemic pressure (clonidine concentration = 0.9–1.4 ng.mL-1). In contrast, very high-dose alpha-2 agonists (clonidine p.o. ≈ 5400–6000 μg/day: plasma concentration = 14–26 ng.mL-1; dexmedetomidine i.v. = 4 μg.kg-1.h-1 for 12 h) evoke hypertension.[210,212] Accordingly, in healthy volunteers, high-dose dexmedetomidine (plasma concentration ≈ 5 ng.mL-1) evokes high BP, lowered ejection fraction and cardiac output.[228] The use of alpha-2 agonists as a bolus or as a loading dose is unwise, especially in unstable CCU patients. Three *different* effects are to be considered. a) A bolus of an alpha-1 agonist (vasopressor : noradrenaline, phenylephrine, etc.) leads to a brisk, short lasting pressure increase[222] via alpha-1 peripheral vascular receptors; the pressure increase stimulates the cardiac parasympathetic system within < 1-2 s[229,230] and generates swift, active bradycardia. This bradycardia is usually not observed in the critical care setting since the cardiac parasympathetic system is wiped out, owing to illness and/or age. b) A *bolus* dose of alpha-2 agonist (dexmedetomidine, clonidine) generates, first, an increase in pressure via activation of peripheral vascular alpha-1 receptors and, second, bradycardia evoked via activation of the cardiac parasympathetic baroreflex (fast active bradycardia), as with any vasopressor. The bradycardia evoked by a pressure increase evoked by a bolus of alpha-2 agonists is exaggerated when parasympathomimetic drugs,[196] opioids[197] or other anti-arrhythmics are present: diltiazem, verapamil, beta blockers and so on. c) *After* waning of the pressure increase evoked by a *bolus* of alpha-2 agonist, a centrally-mediated hypotension occurs once a steady state alpha-2 agonist concentration is achieved (low-dose clonidine concentration). A steady state alpha-2 agonist concentration acts via alpha-2 receptors located in the brain stem and spinal cord, generating a slowly occurring, passive, vascular vasodilation and a slowly occurring, passive, bradycardia.

- **Recommended doses**, titration to effect and changing requirements
  The ‘recommended’ dose for clonidine is 0.2–2 μg.kg.h-1. [131] The recommended dose for dexmedetomidine is 0.4-1 μg.kg.h-1[16] to 1.5 μg.kg.h-1.[12] A ‘ceiling’ effect occurs with dexmedetomidine doses greater than 1.5 μg.kg.h-1.[12] Low-dose alpha-2 agonists include clonidine 0.1–0.4 μg.kg.h-1 and dexmedetomidine: 0.1–0.4 μg.kg.h-1. High-dose alpha-2 agonists (clonidine: 2 μg.kg.h-1, dexmedetomidine: 1.5 μg.kg.h-1) considered here should be differentiated from very high doses of alpha-2 agonists (e.g., clonidine 4 *m*g. kg-1.h-1), which increase blood pressure, as discussed in the legend of Figure 3.
  [A ‘sympathico-meter’ (Ghignone, personal communication) is elusive: plasma catecholamines, sympathetic microneurography or heart rate variability indices8Parasympathetic indices (pNN50; Poincaré plot: SD1 vs. SD2; low-frequency/high-frequency ratio,etc.). In the present manuscript, normalization of sympathetic hyperactivity toward pre-disease baseline activity is emphasized. However, cardiac parasympathetic activity predicted performance in long-distance and ultra-long-distance runners[134,57] and is absent in brain-dead patients. At variance with the present emphasis on the sympathetic system, parasympathetic future research should consider recovery of cardiac parasympathetic activity in CCU patients, for example, following improved conditions and/or administration of alpha-2 agonists.[132] are not yet available online and have not been properly validated in the CCU setting. Therefore, *the dose should be titrated to achieve the required effect* (measured sympathetic or parasympathetic activity or behaviour/sedation, i.e., sedation vs. circulation vs. ventilation vs. inflammation) and adjusted at least daily, rather than administering a ‘recommended’ dose.]

1. **Sedation**
  *Light sedation*: Most groups use dexmedetomidine 0.7 μg.kg-1.h-1 to achieve calmness or light sedation (-2 < RASS < 0),[74] for example, adaptation to NIV,[29] or in a thoroughly stabilized patient. The use of dexmedetomidine is relevant *immediately* after stabilizing the acute cardio-ventilatory distress observed upon admission.
  [In contrast, *heavy sedation* can be achieved by high-dose alpha-2 agonists (clonidine 2 μg.kg-1.h-1 or dexmedetomidine 1.5 μg.kg-1.h-1) as a single agent. High dose alpha-2 agonists will be combined with neuroleptics in the setting of refractory DT (-3 < RASS < -2 for 3–6 h; part II, I, E).[15,17] : alpha-2 agonists, by themselves, are also or combined to neuroleptics, are also applicable in the setting of acute cardio-ventilatory distress upon admission to the CCU[21,81,89,90,135, 136, 137] when neuromuscular blockers for a *short* period suppress the work of breathing, and in the presence of ventilator-to-patient dys-synchrony [3, 4, 5, 6, 7, 8, 9, 10, 11, 12 h: note 14].
  In the setting of moderate/severe ARDS evoked by the SARS-CoV2, they can be safely used, with or without neuroleptics:
  a) with intubation, fever control, optimisation of the acidosis/ cardiac output/microcirculation/acidosis, low pressure support-high PEEP and upright position,[138] b) with high oxygen flow in the setting of non-invasive ventilation, with impressive results (Longrois, unpublished data), following previous reports[29, 30, 31, 32, 33,89]
2. **Circulation**
  *[Hyperdynamic state* (hypertension, tachycardia) without hypovolemia or shock: Dexmedetomidine 1.5 μg.kg-1.h-1 or clonidine 2 μg.kg-1.h-1 is used for as long as necessary. In *rare* instances of refractory hypertension and tachycardia in young patients in the CCU, high-dose alpha-2 agonists should be used first. Then beta-blockers should achieve 50 < HR < 60. Then angiotensin antagonists should be preferred to calcium antagonists or peripherally acting agents to lower BP, to avoid reflex tachycardia.
  *Shock state*

- Systemic circulation (decreased aortic impedance, improved diastolic compliance, reduced sinus or supraventricular tachycardia): Doses as high as dexmedetomidine 1.5 μg.kg-1.h-1 or clonidine 2 μg.kg-1.h-1 can be used only if doses are incremented and volemia is adjusted (‘*start slow and go slow*’: part I, III, F). Presumably, much lower doses normalize tachycardia (part I, III, C).
- Improved microcirculation and tissue acidosis: In the setting of septic shock, after normalization of cardiac output and systemic pH, the issue is restoring microcirculation. [139,140] One of the only tools available is normalizing sympathetic hyperactivity back to baseline. Presumably, lower doses of alpha-2 agonists are necessary to suppress peripheral shutdown and mottling, normalize capillary refill time and lower vasopressor requirements.[38, 39, 40,141]
- Decreased inflammation[39,124,126,127]: Dose-range studies should delineate the dose range as a function of the amplitude of the inflammatory response.]

The dosage used as a rule of thumb in the setting of circulatory instability (e.g., dexmedetomidine 0.7 μg.kg-1.h-1[40] or clonidine 1 μg.kg-1.h-1[38]) is tentative. First, the dosage is a function of maintained[40] stroke volume. Second, the dosage must be documented in a dose-response manner with respect to the pathology and its severity and the considered target: sedation vs. systemic circulation vs. microcirculation vs. inflammation.

B) Clinical reasoning for the use of low- vs. high-dose alpha-2 agonists

*Rationale for high-dose alpha-2 agonists combined with other sedatives*

GABA enhances tonic inhibition in the amygdala. Alcoholism can be related to lowered GABA clearance. GABA-B receptor agonists (baclofen, sodium oxybate) might lower GABA extracellular concentrations, presumably alleviating alcoholism.[142] In the setting of refractory DT, two issues require consideration:

- Psychiatrists observed early that there is no fixed dose of neuroleptics to alleviate refractory DT. Indeed, the dose is the dose necessary to stop agitation and tremor completely, unless exhaustion and death would occur (Quintin, unpublished data); and
- A *combination*9Polypharmacopeia (alpha-2 agonists, neuroleptics, benzodiazepines, melatonin/hydroxyzine, etc.) can cause deleterious interactions. Thus, polypharmacopeia is used for the shortest time possible. of drugs acting via *different* receptors (alpha-2, dopamine, GABA, and glutamate) can help to manage refractory DT.[143] Indeed, refractory DT might be the consequence of excessive central noradrenergic, glutamatergic and hyperactivity. Conversely, reduced cholinergic activity or desensitized GABA receptors might be involved. Alpha-2 agonists are supplemented with neuroleptics if necessary (part II, I). In turn, neuroleptics are supplemented with GABA agonists: low-dose benzodiazepines and/or low-dose baclofen.[144] The objectives are light sedation rendering a rousable patient (-2 < RASS < 0), the absence of muscular tremors, good urine output and minimized side effects (no respiratory depression, long QT, hypotension or bradycardia). As suggested for benzodiazepines when managing alcohol withdrawal syndrome,[143] adequate doses of alpha-2 agonists should be used to maintain *light* somnolence (-2 < RASS < 0) for the duration of delirium. Accordingly, the alpha-2 agonist dose is matched with the desired degree of circulatory effects (tachycardia vs. hypertension vs. microcirculation, etc.) or sedation. Therefore, in contrast to French guidelines[131] and considering this titration to effect, we increased the doses.
  - Clonidine up to 4 μg.kg.h-1 in some elderly patients to achieve calmness, which caused no side effects. Young, combative, patients suffering from poly-addiction (alcohol, cannabis, tobacco, cocaine) seem more responsive to alpha-2 agonists than elderly patients10Elderly humans present with reduced cardiac parasympathetic activity. Alpha-2 agonists increase cardiac parasympathetic activity[145]: any relationship between reduced sedative effects of alpha-2 agonists and reduced central cholinergic or baseline cardiac parasympathetic activity is unknown. Conversely, a) Balinese massage the carotid sinus to induce sleep,[146] b) carotid sinus nerves stimulation tend to make the patient fall asleep (J Karemaker, personal communication). (Simonet, de Kock, Pichot, Quintin, unpublished observations).
  - Clonidine up to 4 μg.kg.h-1 combined to loxapine 400 mg*4 times per day in refractory DT patients presenting with a Gayet-Wernicke syndrome (Pichot-Quintin, unpublished data). Doses as high as clonidine 2700 μg (up to ≈ 38 μg.kg-1 administered over ≈ 30 min) normalize heightened VO_2_ in the setting of weaning from the ventilator and alcohol withdrawal (Figure 2 in [92]). Accordingly, clonidine p.o. up to 3600 μg was used in the setting of ambulatory hypertension.[147] In a medical CCU setting, dexmedetomidine up to 2.5 μg.kg.h-1 was used with minimal side-effects.[12]
- Adaptation of dose of alpha-2 agonists over time

1. **Changing requirements over time**
  The dosage of alpha-2 agonists is not steady throughout the CCU stay for one patient. The dose requirement is very high during early refractory DT to abate agitation and tremors at once for a brief interval (≈ 3–6 h). Nevertheless, drug requirements subside over time.[143] [In the medical CCU setting, we observed a two-phase response11CCU tachycardia is a function of *different* factors:
  - pain;
  - agitation vs. sedation;
  - temperature[90];
  - cardiac baroreflex-mediated mechanisms, which are managed with adequate volemia and increased blood pressure using vasopressors, when necessary;
  - metabo-reflex[148] mediated mechanisms (the metabolic reflex originating in skeletal muscle is ‘*activated when blood flow to contracting muscles is insufficient to allow both O2 delivery and metabolite washout*’; optimized micro-circulation with an alpha-2 agonist could possibly lead to improved tissue acidosis and hypoxemia, presumably reducing tachycardia); and
  - inflammation, which generates tachycardia that is unblocked by a beta-blocker.[149]
  Thus, *tachycardia is partially independent of the sympathetic system*. The clinically unproven implication is that the overall state-of-the-art treatment normalizes inflammation and suppresses tachycardia over a few days. Thus, a high-dose alpha-2 agonist, which controls sympathetically mediated tachycardia, is necessary early to manage inflammation and sympathetic hyperactivity. Subsequently, the high-dose alpha-2 agonist can become detrimental, leading to bradycardia and low cardiac output. Therefore, the doses of alpha-2 agonists must be reduced over time as a function of normalized inflammation. with obvious clinical improvement, e.g. 24-96 h after the beginning of the administration of alpha-2 agonists. Again, the dose of an alpha-2 agonist is titrated to achieve the desired effect.: first, adequate sedation with normalized tachycardia was observed within a few hours. After 24–72 h, bradycardia (40–60 beats per min [bpm]) can occur with possibly an enlarged arteriovenous difference (decreased superior vena cava S_O2_), requiring a reduction of the dose of the alpha-2 agonist to achieve an HR ≈ 60–70 bpm or adequate SsvcO_2_. If the dose of alpha-2 agonist is decreased, then supplementation with neuroleptics might be needed or the dose of neuroleptics increased for a brief period to maintain -2 < RASS < 0, for example, in the setting of refractory DT.]
2. **Changing sedative efficacy**
  In the ambulatory setting, a reduced sedative effect is observed after 2–4 weeks of administration of alpha-2 agonists. [147,150] In the CCU, this reduced sedative effect is observed after a few days (Quintin, unpublished data). The reasons are unclear. They might include improved cerebral perfusion, reduced septic confusion or tachyphylaxis in relation to the sedative effect, warranting a higher dose of alpha-2 agonist or supplementary drugs if required. Therefore, iterative, daily reassessments are necessary.
3. **Changing the required effect from the physician perspective**
  When addressing the acute cardio-ventilatory distress observed upon arrival to the CCU, stabilization of the acute cardio-ventilatory distress is a priority (‘salvage therapy’, ‘resuscitation’). [The sedation requirement is minimal during cardio-ventilatory stabilization. The patient is often unconscious during the acute period (low cerebral perfusion in the setting of hypovolemia; confusion secondary to sepsis). In contrast, as a patient improves, the alpha-2 agonist requirement increases (Quintin, unpublished data). The patient’s comfort is prioritized after the completion of salvage therapy. Titration is performed to the required effect (cognition vs. circulation vs. ventilation vs. inflammation), with the requirement changing over time accordingly.]
4. Interactions with other CCU sedatives
  [Since alpha-2 agonists evoke analgesia[76] and analgognosia,[10] pain in a conscious, alert patient without respiratory depression is treated with nonopioid analgesics as first-line agents (opioid-free analgo-sedation) as in the setting of opioid-free anaesthesia. ‘Rescue’ opiates[151] are prescribed only as a second-line option (part II, tables 1 and 2).]
  If neuroleptics are necessary to supplement alpha-2 agonists, *torsades de pointe* can occur. Thus, the QT interval must be monitored. However, the rare occurrence of QT syndrome should be balanced with the mortality observed in the setting of alcohol withdrawal (part II, I; no delirium: 0–15%; delirium: 34%[152]).

**Table 1:** Delirium Tremens (DT)

Positive diagnosis	History of chronic alcohol intoxication and alcohol withdrawal, inability to sustain attention, disorganized thinking, hallucinations, agitation, fine tremor.
Differential diagnosis	Confusion due to sepsis (beware of occurrence of sepsis or septic shock immediately after resolution of DT or simultaneous to DT), metabolic abnormalities, physical/neurological examination.
Overall assessment	Circulation: iterative response to passive leg raising (PLR) if arterial line in place: normalize volemia before administration of alpha-2 agonists
	Ventilation: ‚focal’ pneumonia?
	Kidney, liver, pancreas, metabolism
	Consider BIS or equivalent when benzodiazepine or propofol infusion are used.
Supportive treatment	Ventilation: O2 supplementation
	Hydration: consider hyperthermia and agitation to evoke adequate diuresis (> 1 mL.kg.d-1, i.e., > 1700 mL/70 kg/24 h)
	Vitamins (B1, B6), nicotine patch(s), eu-glycemia, trace elements, phosphorus, magnesium, calcium supplementation, anti-infectious therapy, if appropriate
	Prophylaxis of thrombosis and gastro-intestinal hemorrhage
	Daily monitoring of K+, Mg++, Phosphorus, Calcium
Sedation	Goal: quiet patient (-1 < RASS < 0): no brisk movements, hallucinations and fine tremor controlled > 24 h
	1) Discontinue benzodiazepine, opioid analgesics and so on immediately upon admission. According to our clinical experience, use benzodiazepines or opioid analgesics only as ‚rescue’ sedation or ‚rescue’ analgesia.
	2) Continue neuroleptics to avoid bout of abrupt agitation upon benzodiazepine/opiates cessation then suppress neuroleptics to make treatment as simple as possible. Would breakthrough occurs, consider haloperidol 5-10 mg i.v. as a “stat” prescription.
	NB: monitor QT when administering any neuroleptics
	Only when alpha-2 agonists are NOT sufficient to evoke -1 < RASS < 0, supplementation with second-line drugs, consider:
	Use preferably haloperidol when hallucinations are the primary symptom, phenothiazines or oxazepines when agitation is the primary symptom.
	a) Loxapine: start using 100 mg orally or through nasogastric tube every 6 h then de-escalate as early as possible down to, for example, 25 mg*4: the goal is -1 < RASS < 0.
	b) Haloperidol 5 mg every 6 h (20 mg/day) or preferably continuous infusion: 50 mg/48 ml/ 0.5–1 ml.h-1 (i.e., start with circa 12–24 mg/day) adjusted to -1 < RASS < 0
	NB: maximal recommended dose for haloperidol : ≈ 30 mg.day-1 (Carrasco, 2016).
	3) Alpha-2 agonists: contra-indication: sick sinus, A-V block II-III, hypovolemia
	Non-intubated patient with some cooperation : clonidine p.o. 6–8 μg.kg-1 (3–4 pills/vials in Europe; beware of various dosages: 1 pill/vial=150 μg in Europe; 1 pill = 100 or 200 or 300 μg in the US) every 4 to 6 h to be administered orally up to 2–3 μg.kg.h-1 for 48–96 h (no tremor > 24 h).
	Suppression of agitation following administration of oral clonidine occurs usually within 30–120 min. This should not imply discharging the patient within 24 h from intermediate care unit: the patient should remain in the intermediate care unit and administered with alpha-2 agonists for 48–96 h (absence of tremor > 24 h) to avoid a second bout of DT after being discharged from the intermediate care unit back to the ward.
	Intubated-mechanically ventilated patient: dexmedetomidine 1.5 μg.kg.h-1 or clonidine 2 μg.kg.h-1 (beware of dosages: Europe: 1 vial =1 mL = 150 μg.mL-1; US epidural clonidine: 100 or 500 μg.mL-1) for 48–96 h adjusted to -2 < RASS < 0; no loading dose: use rescue midazolam (3–5 mg to be repeated) during the interval necessary for alpha-2 agonists to induce ‚cooperative’ sedation (30–60 min for dexmedetomidine; 3–6 h for i.v. clonidine).
	Insert a sticker ‚DO NOT BOLUS’ on the i.v. line (Shehabi 2010).
	Surprisingly, some elderly patients require higher doses of alpha-2 agonists to achieve quietness; by contrast, most young patients, including those on cannabis, heroin, cocaine and so on appear quite sensitive to alpha-2 agonist induced sedation.
Night sedation	Preservation of day-night cycle:
	Hydroxyzine 2 mg.kg-1 (≈ 150 mg/70 kg i.v. or p.o.) or melatonin 1–2 mg will evoke sleep (RASS <- 2), early during the night (administration: 9 pm). Propofol infusion is risky (severe hypotension and/or bradycardia, hypoventilation) when alpha-2 agonists are administered.
	NB: beware of acute urinary retention following hydroxyzine in patients without Folley catheter.
Refractory delirium tremens	See table 2.
Rescue sedation	NB: if sedation is not sufficient with the alpha-2 agonist, do no t EVER administer a bolus of alpha-2 agonist: use ‚rescue’ sedation to be repeated if necessary and increase the administration of i.v. continuous dexmedetomidine up to ‚ceiling’ effect (1.5 μg.kg-1.h-1). consider haloperidol bolus if breakthro ugh occurs.
	To avoid making a complex situation more complex, conventional sedation is to be discontinued abruptly. In intubated mechanically ventilated patients, as i.v. dexmedetomidine or clonidine-induced sedation after ≈ 60 to 180 min respectively, ‚rescue’ sedation (midazolam bolus 3–5 mg titrated to effect) is to be administered repeatedly as required until the alpha-2 agonist evokes quietness to -1 < RASS < 0, combined with a neuroleptics, if needed.
	Before nursing, in intubated mechanically ventilated patients, if needed, consider midazolam bolus 3–5 mg titrated to effect.
	Simple but repeated information of the patient regarding his disease and his care is important to minimize emergence delirium.
Tapering sedation	Following control of DT (no hallucinations nor tremor for > 24 h), neuroleptics are tapered. Then alpha-2 agonists are tapered progressively over several days to avoid the rare occurrence of alpha-2 agonist withdrawal.
Extubation	Alpha-2 agonists do not suppress airway reflexes: a) assess ventilation and overall clinical status (circulation, infection, inflammation, etc.); b) taper neuroleptics first; c) reduce administration of alpha-2 agonists to -1 < RASS < 0, then extubation of the trachea, under alpha-2 agonists.
	Following extubation, if needed, continued non-invasive ventilation under continued alpha-2 agonists as indicated by ventilatory status.
Discharge from CCU	Refrain from discharging the patient early to the ward (hallucinations or tremor should be suppressed for > 24 h): unfortunately, alpha-2 agonists are usually withdrawn on the ward with re-introduction of benzodiazepines leading often for re-admission to CCU and re-intubation.

**Table 2:** Refractory delirium tremens

Positive diagnosis	History of chronic alcohol intoxication and alcohol withdrawal, hallucinations, agitation, fine tremor.
Differential diagnosis	Confusion due to sepsis (beware of occurrence of sepsis or septic shock immediately after resolution of DT or simultaneous to DT), metabolic abnormalities, physical/neurological examination.
Overall assessment	Circulation: iterative response to passive leg raising if arterial line in place: normalize volemia before and during administration of alpha-2 agonists.
	Ventilation: ‚focal’ pneumonia?
	Kidney (consider dexmedetomidine if acute kidney injury), liver (consider clonidine if liver insufficiency), pancreas, metabolism.
	Consider BIS or equivalent if benzodiazepines or propofol infusion are to be used.
Supportive treatment	Ventilation: high 02 flow (Optiflow®) or continuous non invasive ventilation (NIV: consider helmet) as soon as quietness is achieved. The tolerance to continuous, 24/24, NIV is excellent under alpha-2 agonist. Alternate High O2 flow and NIV to minimize skin alterations.
	Hydration: consider hyperthermia and agitation to evoke adequate diuresis (> 1 mL.kg.d, i.e., > 1700 mL/70 kg/24 h)
	Vitamins (B1, B6), nicotine patch(es), eu-glycemia, trace elements, phosphorus, magnesium, calcium supplementation, anti-infectious therapy if appropriate.
	Prophylaxis of thrombosis and gastro-intestinal hemorrhage.
	Daily monitoring of K+, Mg++, phosphorus, calcium.
	Should seizures occur, treat accordingly : benzodiazepine (clonazepam 2 mg bolus, Rivotril® as stat treatment) followed immediately by levetiracetam (Keppra®) and phenytoin (Dilantin®) to avoid over sedation with benzodiazepine.
Sedation	Goal: quiet patient (day: -1 < RASS < 0; night: -2 < RASS < 0): no brisk movements, hallucinations and fine tremor controlled for > 24 h.
	1) Discontinue benzodiazepine, hypnotics, opioid and non-opioid analgesics and so on, immediately upon admission.
	2) Continue administering neuroleptics to avoid bout of abrupt agitation upon benzodiazepine/opiates cessation of administration.
	Only when alpha-2 agonists are not sufficient to evoke -1 < RASS < 0, supplementation with second-line drugs, consider:
	a) Core symptom : agitation: loxapine 100 mg*4-6/day through n/g tube adjusted as early as possible to, for example, 25mg*4 to achieve -1 < RASS < 0.
	NB : monitor QT when administering loxapine.
	or cyamemazine (Tercian®) 25 mg*3 up to 50*3
	or levomepromazine (Nozinan®) 50–200 mg/day continuous i.v.
	or chlorpromazine (Largactil®) 50–200 mg/day continuous i.v.
	b) Core symptom : hallucination: haloperidol 5mg every 6 h (20 mg/day) or preferably continuous infusion: 50 mg/48 ml/ 4 mL.h-1 (i.e., start with circa 100 mg/day) adjusted to 25 mg/day to -1 < RASS < 0.
	NB: maximal recommended dose for haloperidol : ≈ 30 mg.day-1 (Carrasco, 2016). De-escalate as early as possible.
	c) Tiapride 100–1200 mg/day : 1200 mg/48 ml 2 mL.h-1 adjusted to -1 < RASS < 0.
	3) Start administering alpha-2 agonists:
	Contra-indication: sick sinus, A-V block II-III, hypovolemia.
	Refractory DT is very rarely managed without tracheal intubation. The usual presentation in the CCU is a patient who has been intubated to allow for conventional sedation (light total intravenous anesthesia, analgo-sedation). In non-intubated patient with some cooperation: clonidine 3–4 pills/vials (1 pill/vial = 150 μg in Europe) every 4–6 h to be administered orally up to 2–3 μg.kg.h-1 for 48–96 h.
	Suppression of agitation following administration of oral clonidine occur usually within 60–120 min. This should not imply discharging the patient within 24 h from critical care unit (CCU): the patient should remain in the CCU and administered with alpha-2 agonists for 48–96 h (absence of tremor) to avoid a second bout of DT after being discharged from the intermediate care unit to the ward.
	Intubated-mechanically ventilated patient: dexmedetomidine 1.5 μg.kg.h-1 or clonidine 2 μg.kg.h-1 for 48–96 h adjusted to -2 < RASS < 0; no loading dose: use rescue midazolam (3–5 mg to be repeated) during the interval necessary for alpha-2 agonists to induce ‚cooperative’ sedation (30–60 min for dexmedetomidine; 3–6 h for i.v. clonidine).
	Insert a sticker ‚DO NOT BOLUS’ on the i.v. line for alpha-2 agonist (Shehbi 2010).
	Some elderly patients require higher dose of alpha-2 agonists (clonidine up to 4 μg.kg.h-1) to achieve quietness; by contrast, most young patients on cannabis, heroin, cocaine and so on (alone or in addition to alcohol) appear quite sensitive to alpha-2 agonist evoked sedation.
	The treatment of refractory DT rests on the association of several drugs (alpha-2 agonists+neuroleptics: alpha-2+haloperidol+tiapride or alpha-2+loxapine+tiapride) to evoke quietness through different mechanisms with minimal circulatory or ventilatory side-effects. As the patient improves, de-escalate drugs as early as possible : suppression of neuroleptics, then of alpha-2 agonists.
	In rare instances, SBP may be low: a) check for etiology (volemia, sepsis, etc.); b) use low dose noradrenaline rather than tapering alpha-2 agonists; c) a second best practice is to lower the dose or suppress alpha-2 agonist administration and carry on with neuroleptics, scaled up to absence of agitation, hallucination, tremor. Basically, there is no maximal dose for neuroleptics: the patient should be quiet without tremor without resorting to general anesthesia or high dose benzodiazepine (ventilatory side-effects).
	In case of Gayet-Wernicke or refractory DT, very high doses of alpha-2 agonists and neuroleptics are needed to achieve quietness and absence of tremor (e.g., clonidine 4 μg.kg.h-1+loxapine 400 mg*4±tiapride). The issue is to clinically overcome agitation, hallucinations and tremor, irrespective of the dose administered, then de-escalate as early as possible (no tremor > 24 h).
Night sedation	Preservation of day-night cycle:
	Hydroxyzine 2 mg.kg-1 (≈ 150 mg/70 kg i.v. or p.o.) or melatonin 1–2 mg (or their combination with lower doses) will evoke sleep, early during the night (administration: 8–9 pm). Propofol or midazolam infusion appear unwise especially in the setting of hypotension or hypoventilation.
	NB: acute urinary retention is a possibility following administration of hydroxyzine in patients without Folley catheter.
Rescue sedation	NB: if sedation is not sufficient with the alpha-2 agonist, do not EVER administer a bolus of alpha-2 agonist: use ‚rescue’ sedation to be repeated if necessary and increase the administration of i.v. continuous dexmedetomodine up to ‚ceiling’ effect (1.5 μg.kg-1.h-1).
	To avoid making more complex a complex situation, conventional sedation is to be discontinued abruptly. In intubated mechanically ventilated patients, as i.v. dexmedetomidine or clonidine evoke sedation after ≈ 60 to 180 min respectively, ‚rescue’ sedation (midazolam bolus 3–5 mg) is to be administered repeatedly as required until the alpha-2 agonist evokes quietness to -1 < RASS < 0, combined with a neuroleptics, if needed. Would breakthrough occurs, consider haloperidol 5-10 mg bolus.
	Before nursing, in intubated mechanically ventilated patients, consider midazolam bolus 3 mg (repeatedly if needed, i.e., titrated to effect) if needed.
	Simple information repeatedly given to the patient regarding his disease and his care is important to minimize emergence delirium.
Tapering sedation	Following control of DT (no hallucinations nor tremor for > 24h), neuroleptics are tapered. Then alpha-2 agonists are tapered progressively over several days to avoid the (rare) occurrence of alpha-2 agonist withdrawal.
Extubation	a) Assess overall clinical status (ventilation, circulation, infection, inflammation, etc.); b) taper neuroleptics first; c) reduce administration of alpha-2 agonists to -1 < RASS < 0, then extubation of the trachea, under alpha-2 agonists: alpha-2 agonists do not suppress airway reflexes.
	Following extubation, continued NIV and/or Optiflow® under continued alpha-2 agonists as indicated by ventilatory status.
Discharge from CCU	Refrain from discharging the patient early to ward (no hallucinations nor tremor for > 24 h): alpha-2 agonists are usually withdrawn on the ward with re-introduction of benzodiazepines leading often to re-admission to CCU and re-intubation.

**1) Rescue sedation**

- **Facilitation of nursing**
  Handling patients -4 < RASS < -3 vs. patients -2 < RASS < 0 requires a *Copernican* revolution in the CCU (part I, II). Accordingly, the use of rescue sedation[74] allows to keep a patient under light sedation throughout most of his stay (-2 < RASS < 0) and to implement deeper sedation for a few minutes to facilitate nursing procedures.
- **Rescue sedation upon or before initiation of alpha-2 agonist administration**
  When switching from conventional sedation to cooperative sedation, the possibility is either:
  - a slow, even, transition, for example, when an elderly patient has received high-dose midazolam and sufentanil for days or weeks12An infusion of midazolam 5 mg.h-1 totals circa 120 mg over 24 h, usually for several days. This dosage is a massive overdose for an elderly, frail patient, leading to delayed emergence.; or
  - emergence and agitation, as observed in combative patients as soon as conventional sedation is withdrawn. Such a patient will be likely poorly sedated and agitated as long as steady cooperative sedation has not yet been achieved. Thus, rescue sedation[74] allows one to transition from conventional sedation to dexmedetomidine: midazolam 0.01–0.05 mg.kg-1 (1–3.5 mg/70 kg) is administered at 10 to 15-min intervals to achieve -2 ≤ RASS ≤ -1.[74] [In our group, midazolam boluses of 3–5 mg are repeated every 3–5 min up to -2 ≤ RASS ≤ -1 until steady cooperative sedation is achieved (≈ 60 and 180 min for dexmedetomidine 1.5 μg.kg.h-1 or clonidine 2 μg.kg-1.h-1, respectively). Under extreme circumstances (e.g., DT with refractory agitation), neuroleptics are administered *before* transitioning from conventional to cooperative sedation (haloperidol 2.5–10-mg bolus or loxapine 100 mg through a nasogastric [n/g] tube). Then, conventional sedation is withdrawn, and cooperative sedation is administered.]
  Progressively withdrawing conventional sedation while gradually introducing an alpha-2 agonist[16] leads to severe bradycardia/hypotension (summation of sympatholysis), in our hands. This occurs often at night if the night shift personnel has not been taught about using rescue sedation rather than propofol boluses or infusions. There is *no* rationale for running conventional sedation in addition to alpha-2 agonist administration, or the reverse, but a recipe for circulatory catastrophes. In addition, running conventional sedation in addition to cooperative sedation suppresses any possible benefit of cooperative sedation.[153] The use of a propofol bolus (mini-dose: 20 mg)[16] or propofol infusion, in addition to alpha-2 agonist administration during the night presumably reflect the practice of an experienced intensivist working with experienced critical care nurses.[16] In our hands, this practice increases the risk of *unexpected severe arterial hypotension and bradycardia* at night. Then, the alpha-2 agonist is blamed, leading to a switch back to conventional sedation, CMV, NMBs, and high-dose vasopressors.]
- **Night sedation**
  The depth of sedation in septic patients may be varied during the day-night cycle (day time: -1 < RASS < 0; night time: -2 < RASS < 0).[27] In the CCU setting, increased sedation at night (-2 < RASS < -1; medium-dose dexmedetomidine: 0.4–0.7 μg.kg.h-1 or low-dose dexmedetomidine: 0.1 μg.kg.h-1) reduces the fragmentation of sleep and increases sleep stage 2 (N2) without affecting REM sleep.[71,154] In healthy volunteers, low-dose clonidine (25 μg) increases the REM sleep duration.[69] By contrast, medium-dose clonidine (150 μg) lowers the REM sleep duration and increases the slow-wave sleep duration.[69] [In our experience, once steady cooperative sedation has been achieved with an alpha-2 agonist alone or in combination with neuroleptics, our prescription regimen maintains the alpha-2 agonist at a steady dose. To deepen night sedation, if necessary, administration of melatonin provides good clinical results (administration ~ 8–9 pm considering its delayed effect; 2 mg orally or through an n/g tube). However, the results are equivocal.[155,156] Alternatively, hydroxyzine 100–150 mg administered i.v. over 10 min or orally through an n/g tube achieves early awakening the next day without delayed somnolence. A combination of these drugs at lower doses may also be used.]
- **Monitoring**
  To avoid oversedation, electroencephalographic monitoring (BIS or equivalent) is advocated when deep sedation is required (NMB use, status epilepticus, etc.).[5] To our knowledge, BIS monitoring under cooperative sedation has been rarely reported. A sedation scale ‘to effect’ verified iteratively should be preferred to a tentative 60 < BIS < 95 throughout a patient’s stay[157] as the issue is not the displayed BIS value but the actual sedative effect.
- **Bronchospasm and circulatory instability [1) Bronchospasm**[31, 32, 33,87]:
  When, severe, prolonged bronchospasm or status asthmaticus are considered, *despite* optimal management, a low-dose alpha-2 agonist (inhaled[85] or intravenous clonidine 4–9 μg.kg-1 over 2–4 h[31] or dexmedetomidine 0.2–0.7 mg.kg-1.h-1[32,33] suppresses expiratory obstruction (wheezing, forced expiration, ventilatory discomfort) and agitation within ≈ 60–120 min. However, when presented with aversive psychological stimuli, relatives and so on, some patients require a high-dose alpha-2 agonist (clonidine 2 μg.kg.h-1, dexmedetomidine 1.5 μg.kg.h-1) titrated to effect (quietness and the absence of wheezing even when an aversive stimulus is presented), as in refractory DT. Failure to achieve quietness and suppressed wheezing warrants intubation, sedation and conventional mechanical ventilation before ventilatory fatigue.

**2) Circulatory instability**

Addressing the indication and dose of alpha-2 agonists in the setting of elderly patients, co-morbidities, shock and peripheral shut-down (mottling) is complex. Our practice is threefold:

- Iterative individualized volume loading, based on iterative passive leg raising (PLR; Figure 6) and echocardiography. [158]
- Restoration of systemic pressure with vasopressors when diastolic pressure is low.[159] In this respect, alpha-2 agonists *lower* the dose of vasopressor required in the setting of sepsis[25,160,161] or septic shock,[38, 39, 40] in a dose dependant manner,[162] within ≈ 3 h of administration (Pichot, unpublished data).[38,39]
- Restoration of the microcirculation[139] in the setting of peripheral shut-down, increased lactate, etc.13Patients with a low temperature in the setting of sepsis present higher mortality[163,164]: they are either unable to generate ‘core’ heat or to transfer ‘core’ heat to the periphery. Peripheral shutdown could be a cause or consequence of this inability. Few pharmacological tools address peripheral shutdown[114,165]: alpha-2 agonists increase lactate clearance,[166] lower systemic lactate[167,168]), temperature[88] and VO2,[91] improve capillary refill time and kidney function.[119,120]

F) Speed of administration

1. ***De novo* administration in a circulatory-stable patient**
  Before administering an alpha-2 agonist, physical examination, benefits/risks, bradycardia, sick sinus syndrome, A-V block, and hypovolemia should be assessed. [In the setting of alcohol withdrawal/agitation, *without* hypovolemia, an alpha-2 agonist is administered at a fixed dose (e.g., dexmedetomidine 1.5 μg.kg-1.h-1 or clonidine 2 μg.kg-1.h-1) to achieve quick sedation. Rescue sedation is administered to RASS ≤ 0, if needed (part I, III, D).]
2. ***De novo* administration of alpha-2 agonist in the setting of hypovolemia**
  CCU patients are often hypovolemic. Hypovolemia becomes apparent emerges when sedation (conventional or cooperative) is administered (part II, insert II) and controlled mandatory ventilation is initiated with positive end-expiratory pressure (PEEP). Therefore,
  - Pre-emptive volume loading is wise when CMV is anticipated.[169]
  - Volemia should be assessed and corrected appropriately[158] before initiating the administration of an alpha-2 agonist (e.g., iterative PLR[170] [Figure 6]; transthoracic echocardiography [TTE]: ventilation-induced changes in the inferior vena cava[171]; transoesophageal echocardiography [TEE]: collapsibility of the superior vena cava[172]). Capillary refill, diuresis, the CO_2_ gap, the lactate concentration, and the arterio-venous difference should be monitored before any further increase in the speed of administration of the alpha-2 agonist up to the required effect (sedation vs. circulation vs. ventilation vs. inflammation).
  - ‘*Start low and go slow*’14A ‘*start low and go slow*’[173] approach introduces a beta blocker in the setting of very severe left ventricular failure, outside the CCU setting. Similarly, a ‘*start slow and go slow’* approach summarizes, in our hands, the administration of an alpha-2 agonist when acute cardio-ventilatory distress is present, within the CCU setting.: Greater hypovolemia warrants a lower speed of administration, for example, dexmedetomidine 0.125 μg.kg-1.h-1 for 2–3 h, followed by TTE+PLR reassessment and then 0.25 μg.kg-1.h-1 for 2–3 h, followed by TTE+PLR, and so on, up to the effect or to 1.5 μg.kg-1.h-115Given the high bioavailability of oral clonidine, slow loading is also possible through an n/g tube, for example, clonidine 150 μg crushed and administered through the nasogastric tube. Then, 2 h later, after PLR and ETT re-assessments, clonidine 300 μg can be administered through the n/g tube iteratively until the desired effect is achieved, followed by i.v. administration of the required dose. This route of administration appears suitable in mechanically ventilated patients as a bridge (12–24 h) to i.v. administration; then, gastric accumulation occurs with possible bradycardia (Quintin, unpublished data). (part I, III, B,: here, the goal is improved microcirculation or reduced vasopressor requirement not sedation: set -2 < RASS < 0). An interval of 6–12 h from admission to achieve administration of the full dose of an alpha-2 agonist and steady cooperative sedation is acceptable since the patient will be staying in the CCU for days. Rescue sedation should be immediately available (part I, IV). Sympatholysis evoked by the alpha-2 agonist progressively increases the venous capacitance *before* leading to brisk arterial hypotension (part II, insert II). In addition: a) diaphragmatic paralysis suppresses the inspiratory squeezing of the blood expelled from the hepato-splanchnic sphere into the thorax[174]; and b) intrathoracic pressure increases secondary to positive pressure ventilation. Then, circulatory failure occurs because of vena cava collapse (part II, insert II). Thus, assessing volemia is important *before and early* during administration. Compression stockings or anti-shock trousers are to be considered.]
3. ***De novo* administration of an alpha-2 agonist in the presence of vasopressor**
  Vasopressors squeeze the venous capacitance.[175] In contrast, alpha-2 agonists inhibit the vasomotor sympathetic activity targeted to the veins. Therefore, iterative assessments of venous return and replenishment of the venous capacitance will avoid: a) lowering venous return any further *before* each incremental increase in an alpha-2 agonist (part I, III, F); andb) arterial dilation.
4. **Switch from conventional to cooperative sedation**
  When conventional sedation is used prior to an alpha-2 agonist, the benefits/risks should be assessed, as above. [Withdrawal of conventional sedation is performed *at once* (part I, III, D), and an alpha-2 agonist infusion is initiated. A high dose (dexmedetomidine: 1.5 μg.kg-1.h-1; clonidine: 2 μg.kg-1.h-1; part I, III) will be selected as a function of the desired effect, for example, quick implementation of sedation in refractory
  DT. In contrast, a low-dose alpha-2 agonist will be selected (dexmedetomidine or clonidine: 0.125 or 0.25 μg.kg-1.h-1 for 3 h, followed by an increasing dose when shock or circulatory instability is present (part I, III, A).] Rescue sedation[74] is administered repeatedly if agitation occurs before achieving steady cooperative sedation (part I, III).
  [When high-dose dexmedetomidine or clonidine does not achieve quietness (-2 < RASS < -0) within 3 and 6 h, respectively, neuroleptics should be added according to the primary symptom (hallucination: haloperidol vs. agitation: loxapine; Pichot, personal communication; part II, I, E). Benzodiazepines should not be used except for rescue sedation, anxiolysis and emergency treatment of seizures. Midazolam infusion set minimally (e.g., 0.5 mg.h-1) may be considered only if anterograde amnesia is a concern, for example, with a patient in the prone position under short-term neuromuscular blockade requiring deep sedation (RASS < -2) *after* documentation of hypnosis (40 < BIS < 60) and analgesia (BPS), under high-dose alpha-2 agonist supplemented first with neuroleptics).]
5. **Switch to spontaneous breathing**
  The switch to spontaneous breathing should occur as soon as cardio-ventilatory distress has improved,[21,90,176] regardless of selecting conventional vs. cooperative sedation. *Alpha-2 agonist administration following light TIVA*: Most intensivists use alpha-2 agonists as second-line sedatives, that is, during recovery after switching from conventional sedation. Deep sedation (i.e., general anaesthesia) is required to manage severe acute respiratory distress syndrome (ARDS), traumatic brain injury, septic shock[177] and status epilepticus: this *unproven*[178] belief has been challenged.[179,180] [When severe diffuse ARDS is managed under general anaesthesia, paralysis,[181] and prone positioning,[182] the patient is positioned with the head up[183] (“upright” position) and then switched to an alpha-2 agonist. Conventional sedation is withdrawn immediately up to steady cooperative sedation used alone (part I, III, D). Then, spontaneous breathing is resumed.]
  *[Alpha-2 agonist administration immediately following endotracheal intubation*: In the setting of early severe diffuse ARDS, we do not use midazolam+sufentanil or propofol+remifentanil.[21,89,90] To reduce the duration of mechanical ventilation and intubation,[37,184] we administer an alpha-2 agonist *immediately* after intubation to achieve an -2 < RASS < 0. A neuroleptic is added to a RASS < -2, *if* needed. Ultra-short-term paralysis (3–12 h, 40 < BIS < 60) is used only to allow for normalizing all of the factors evoking a high respiratory drive in an itemized manner.[21]16Briefly: fever control,[90] suppressed agitation,[21,89,135,136, 185,137] normalized systemic pH,[136] upright position,[183] a low ‘CO_2_ gap’,[186] normalized lactate, normalized superior vena cava O_2_ saturation, minimal permissive hypercapnia[187] [as during physiological stage 2 sleep, a lowered sensitivity to CO_2_ is observed following alpha-2 agonist administration[88]], a normalized metaboreflex (note 11),[148] normalized tissue pH and improved capillary refill, reduced inflammation, reduced lung water.[188] As soon as the acute cardio-ventilatory distress is controlled,[21,90,176] paralysis is withdrawn. Then, spontaneous breathing allows one to set a high PEEP with a low plateau pressure[21,81,89,135,136,185] to address early expiratory closure (ARDS with low VA/Q ratio, at variance with COVID 19-ARDS) and hypoxemia. The work of breathing is minimized with a low level of inspiratory assistance to compensate for the circuit, valves and endotracheal tube using pressure support or airway pressure release ventilation.]
6. **Tapering of alpha-2 agonists**
  Clonidine[189] or dexmedetomidine withdrawal syndrome is rare. [To avoid alpha-2 agonist withdrawal syndrome, when a high-dose alpha-2 agonist has been administered for an extended period, a short-acting alpha-2 agonist is transitioned to a longer-acting alpha-2 agonist: intravenous dexmedetomidine (t1/2β ≈ 180 min) is transitioned to oral clonidine, which is then discontinued over several days (clonidine: t1/2β ≈ 8-–24 h[48,190]; clonidine 0.2–0.5 mg every 6 h and then every 9 h, and so on, over 1–7 days; median: 2.4 days[191]). Similarly, i.v. clonidine is transitioned to oral clonidine or oral guanfacine (extended release Intuniv®, Estulic®, t1/2β ≈ 10–30 h). Where available (e.g., the US), transdermal clonidine (Catapres-TTS® 7.0 or 10.5 cm^2^) can be considered as soon as capillary refill is normalized.]
7. **Discharge to the ward**
  [If *progressive* tapering of an alpha-2 agonist is not guaranteed in the ward, then the patient should stay in the intermediate care unit. Premature discharge to the ward can lead to later readmission to the CCU. Accordingly, when DT is improving, the patient should remain in the intermediate care unit to assess positively the total disappearance of tremors and hallucinations for ≥ 24 h.]
  G) Side effects
  The side effects of alpha-2 agonists[192] are a function of the: a) adequacy of venous return (part II, insert II); and b) adaptation of the cardiac and vasomotor limbs of the baroreflex (legend of Figure 1):

1. **Circulation**
  Clonidine upregulates beta receptors in healthy supine volunteers.[193] Clonidine is used in the setting of severe idiopathic orthostatic hypotension because it increases blood pressure[194] by upregulating alpha-1 receptors.[141] When p.o. clonidine is administered to normotensive or hypertensive patients without idiopathic orthostatic hypotension, systemic hypotension (SBP < 100 mm Hg) is seldom observed. When systemic arterial hypotension occurs, the patient seems unaffected (adequate capillary refill, preserved urine output). Nevertheless, since mild hypotension is not tolerated in the ward, the patient should stay in the intermediate care unit.
  [Adequate volemia and atrio-ventricular conduction should be achieved before infusion of alpha-2 agonists (part I, III, F). When a continuous i.v. infusion of alpha-2 agonists is used, hypotension+bradycardia is observed in 5–33% of patients. [12,195] Management of side effects includes:
  - Avoiding boluses or a loading infusion (a sticker stating ‘DO NOT BOLUS’ is placed on the i.v. line infusing the alpha-2 agonist[16]);
  - Increasing venous return (stockings/military anti-shock trousers, Trendelenburg position, volume loading) before the administration of alpha-2 agonist;
  - Avoiding drugs that increase parasympathetic activity, leading to severe bradycardia and hypotension, for example, opioids[196,197];
  - Avoiding drugs that suppress cardiac or vasomotor sympathetic activity, for example, beta-blockers and propofol; and
  - Administration of micro-doses of norepinephrine (infusion: 0.01–0.05 μg.kg-1.min-1)[195] when urine output or peripheral or cerebral perfusion is a concern.
  *Interactions between antiarrhythmics and alpha-2 agonists*: All antiarrhythmics can be used with alpha-2 agonists. However, as a rule of thumb, lowered dosages and a reduced speed of administration should be considered with continuous P-R monitoring. For example, amiodarone (Cordarone®) administration is decreased over 2–3 days: a high dose (900 mg/ day) without co-administration of alpha-2 agonists is reduced to a low dose (300 mg day-1) with co-administration of an alpha-2 agonist or preferably to complete cessation, as a function of HR or arrhythmias. Amiodarone boluses will be reduced, especially if hypovolemia or BP instability is present (from 300 mg over 20 min without co-administration of an alpha-2 agonist to 150 mg over 40 min when an alpha-2 agonist has been administered before amiodarone). Verapamil (Isoptine®) can be administered using a 1.25 mg bolus repeated every 2–5 min as required. When severe hypovolemia and bradycardia occur, the Trendelenburg position and immediate administration of CaCl_2_ 1 g and ephedrine 15 mg are applied, followed by brisk volume expansion.]
2. **Ventilation**
  Ventilatory depression does not occur with alpha-2 agonists[41] except in the setting of accidental or intended overdose. The absence of ventilatory depression makes alpha-2 agonists the first-line drug in the setting of non-invasive ventilation[29,30,198] (high flow oxygen, helmet or mask ventilation): could this be extended to proning in awake patients, for example, with respect to the covid-19 pandemics?[199] In the CCU, at the doses described above[12,131] (part I, III), respiratory depression is not observed.[41,47,80,88,200] When high-dose benzodiazepines are administered *before* admission to the CCU, oversedation, respiratory depression,17Midazolam i.v. (2 mg) after oral administration of clonidine (300 μg) leads to transient respiratory depression, rarely (Quintin, unpublished data). In fact, low-dose benzodiazepines administered alone evoke transient respiratory arrest, rarely.[201] or atelectasis can occur. [The options are immediate cessation of benzodiazepine administration (part I, III, D), administration of a benzodiazepine antagonist (flumazenil) if appropriate, administration of continuous infusion of an alpha-2 agonist without a loading dose, ventilatory monitoring and transient NIV or invasive ventilation, if needed.]
  Upper airway obstruction is observed in patients presenting with sleep apnoea[72] and healthy volunteers,[202] presumably during slow-wave sleep (‘snoring’). Whether such obstructions are clinically relevant during NIV requires study.
3. **Temperature**
  Hyperthermia following administration of dexmedetomidine has been reported (n = 9[203]), although alpha-2 agonists act on the hypothalamic activation threshold (‘set point’), generating mild hypothermia (35–35.5°C).[88,94,204,205] [This report contradicts our clinical experience with alpha-2 agonists in facilitating fever control in the setting of septic shock or ARDS.[90]] Nevertheless, hyperthermia should be borne in mind as a possible adverse drug reaction.
4. **Ogilvie syndrome**
  When alpha-2 agonists are used as premedication or within the setting of opioid-free anaesthesia[122] (Quintin, unpublished data), bowel movement is observed before surgery. Clonidine reduces diarrhoea in the setting of rapid opioid detoxification under general anaesthesia.[206] Accordingly, Ogilvie syndrome has been observed immediately following the introduction of alpha-2 agonists[207] in the setting of heavy sedation.[208] Could the pro- or antikinetic overall effect be related to baseline parasympathetic vs. sympathetic dominance?

### IV) Dexmedetomidine vs. clonidine

Medetomidine (racemic of levo- and dexmedetomidine) exhibits an alpha-2/alpha-1 selectivity ratio of 1620; conversely, clonidine has a selectivity of 220.[209] Selectivity for alpha-2 receptors is only *relative*. Furthermore, this selectivity is only an *in vitro* argument, with only marketing relevance and *no* clinical relevance, for differentiating dexmedetomidine from clonidine. Selectivity is relevant only when very high-dose alpha-2 agonists are administered, leading to hypertension[44,210, 211, 212] (discussion in Figure 3). Factually, the pharmacodynamics of dexmedetomidine and clonidine are identical. In addition, no head-to-head comparisons of the 2 drugs have been conducted in various CCU settings (but ClinicalTrials.gov : NCT03653832). Thus, alpha-2 agonist selection is a function of the setting and the patient.

1. Clonidine is available as a p.o., i.v., or transdermal drug only in some countries. Conversely, dexmedetomidine is an i.v. drug, usable as a spray in children.
2. Clonidine is inexpensive and widely available. Dexmedetomidine is more expensive than clonidine; however, the reduced duration of ventilation and length of CCU stay compared to those with conventional sedation[37,83] generate savings.
3. Clonidine is bioavailable with oral (90%)[44] and rectal (95%) administration.[213] Dexmedetomidine is less available when administered orally (15%).[214] Both drugs are well absorbed intranasally.
4. Clonidine is metabolized to inactive metabolites through the liver (50%) and excreted unchanged via the kidneys (50%). [214] Therefore, clonidine is relatively contraindicated in the setting of acute kidney injury. [Nevertheless, since renal replacement therapy (RRT) is available in the CCU, this contraindication is relative. If RRT is used, then the dose of clonidine is usually titrated upwards to achieve the desired effect (Quintin, unpublished data).]
5. Dexmedetomidine is excreted via the liver; therefore, acute liver insufficiency is a contraindication. Alternatively, the dose should be lowered.
6. [In stable patients, sedation is achieved earlier with dexmedetomidine (30–120 min) than with clonidine (120–240 min). Thus, *the nursing staff considers dexmedetomidine easier to use* (Simonet, de Kock, personal communication). Nevertheless, rescue sedation[74] (part I, III, D) is necessary to compensate for the non-instantaneous sedative effect evoked by both alpha-2 agonists. The drawback of the slower effect of clonidine is relative. Since a patient will stay in the CCU for an extended period, a difference of ≈ 2–3 h to achieve a peak sedative effect will not affect the length of stay or costs (steady cooperative sedation with dexmedetomidine: 2–4 h; with clonidine: 3–6 h; Pichot, unpublished data). Furthermore, unstable patients require a ‘*start low and go slow*’ approach, regardless of whether dexmedetomidine or clonidine is used (part I, III, F).]
7. The elimination half-life of dexmedetomidine is ≈ 180 min vs. ≈ 8–24 h for clonidine.[190] A shorter half-life is an advantage if abrupt discontinuation of the alpha-2 agonist is needed and a disadvantage during tapering (part I, III, F).

## Conclusion

Currently, no evidence-based data support any expert-based recommendations or an international consensus regarding the use of alpha-2 agonists in the CCU. Furthermore, no extensive randomized, controlled trials have addressed the use of alpha-2 agonists *specifically* for the pathologies considered here. Thus, despite biases, the present views are valid because they reflect years of clinical practice in different countries. These views emphasize the potential use of alpha-2 agonists as *first-line* sedative agents[15, 16, 17,73,74] in the CCU. We hope that the mistakes that we have made can be avoided in terms of the selection of patients, including frail patients, the speed and dose of alpha-2 agonists according to required effect, the goal (sedation vs. systemic circulation vs. micro-circulation vs. ventilation vs. inflammation), and the use of alpha-2 agonists in combination with neuroleptics and opioid-free analgo-sedation. Clinical decisions should consider the benefit/risk ratio in *each* patient before potent drugs, such as alpha-2 agonists, are administered. A learning curve is suggested: a) first administer alpha-2 agonists in stable patients without hypovolemia (e.g., patients with alcohol withdrawal or refractory DT); and then

b) move toward unstable patients (‘*start low and go slow*’ approach: hypovolemia/hypotension, multiple trauma, etc.) using iterative circulatory optimization. Finally, whether alpha-2 agonists improve,[23, 24, 25, 26, 27, 28] or not,[68] the outcome requires stronger evidence in sick patients, without co-administration of conventional sedation.[153,215] In addition, niche indications are to be considered: one size does *not* fit all. Hopefully, evidence-based data will inform evidence-based international guidelines.

## Part II: Clinical Applications

### I) Delirium

CCU delirium, alcohol withdrawal, established delirium tremens and delirium tremens refractory to haloperidol[200] are considered sequentially.

- **CCU delirium**
  Delirium is characterized by: a) the acute onset of cerebral dysfunction with a change or fluctuation in baseline mental status, b) a reduced ability to focus, shift or sustain attention, a disturbed level of consciousness, or reduced clarity of awareness of the environment, and c) cognitive (disorganized thinking, memory deficits, disorientation, or language disturbance) or perceptual disturbance (hallucinations or delusions).[2] Delirium may be ‘hypo-active’ (66% of CCU patients; inattention, disordered thinking, or a decreased level of consciousness without agitation),[5] for which prognosis is poor.[234] Conversely, delirium may be hyperactive (2% of CCU patients[5]). Delirium is associated with increased mortality, prolonged CCU and hospital stays, and cognitive impairment.[2] CCU mortality increases from 0–15% when delirium does not occur[143] to 34% when delirium occurs[152]: a 10% increase in the relative risk of death for each day of delirium exists (quoted from (234)). Therefore, early diagnosis[130] and treatment of delirium are required. However, evidence linking treated delirium and improved outcomes is missing.[234]
  Before diagnosing nonspecific delirium, other causes should be considered after neurological examination and neuro-imaging: sepsis, pain, hypoxia, hypoglycaemia and hypotension, followed by withdrawal from alcohol, opioids or illegal drugs.
  Current recommendations suggest avoiding benzodiazepines with long terminal half-lives.[4] Benzodiazepines are currently used only as *short-term* medications for co-induction of general anaesthesia, rescue sedation,[74] anxiety, seizures and amnesia. Among ventilated patients (assist control, proportional assist ventilation, pressure control), a reduced incidence of delirium is observed when low-dose dexmedetomidine is administered to prevent delirium as a night-time sedative (RASS=-1, 9 pm–6 am).[235] When opioid doses were unchanged and sedative doses were halved,[235] 80% of patients in the dexmedetomidine group did not present with delirium vs. 54% in the control group. Dexmedetomidine patients are less tired, presumably due to reduced sleep fragmentation.[71]
  *[Extubation and agitation after extubation*: As protective airway reflexes are unaffected following administration of high-dose alpha-2 agonists in the ambulatory cardiology setting,[147] reducing the dose of the alpha-2 agonist before extubation is not necessary. The point is not the dose of the alpha-2 agonist, but the *alertness* of the patient (-2 < RASS < 0) without inappropriate agitation after extubation. Lastly, when an alpha-2 agonist has not been administered prior to extubation, and if the patient becomes agitated after extubation, then an alpha-2 agonist is administered and titrated to achieve calmness after extubation. This eases the management when *continuous* non-invasive ventilation (NIV) is undertaken for several days before intubation or after early extubation.]
- **Prevention of alcohol withdrawal**
  Alcohol dependence is present in 15–20% of all hospitalized patients.[143] During hospitalization, 8–31% of these alcohol-dependent patients develop alcohol withdrawal symptoms (AWS) with neurologic and autonomic dysfunctions. Approximately 15 and 5% of these AWS patients will develop tonic-clonic seizures or hyperactive DT (agitation, delirium, seizures, hypertension, tachycardia, arrhythmia, hyperpyrexia, diaphoresis), respectively.[2]
  Conventional prevention of DT includes identification and monitoring of patients at risk, early mobilization, cognitive stimulation, physiotherapy, noise reduction, a preserved day-night cycle, vision and hearing, continued orientation and information, ample hydration,[234] and vitamins (B1: thiamine; B6: pyridoxine). [Pre-emptively treating a patient pharmacologically before overt DT may lead to overtreatment of patients who will not develop DT. As long as a patient is continuously scrutinized in the intermediate care unit, monitoring the Confusion Assessment Method for the ICU (CAM-ICU) score, hallucinations, agitation and tremors is the deterrent to the development of DT.]
- **‘De novo’ alcohol withdrawal**
  1. **Admission from the ward**
    Patients in the ward or the CCU/intermediate care unit are often agitated, under physical restraint and receiving intermittent doses of benzodiazepines i.v. or p.o. (Table 1). [However, they retain some coherence for a few seconds or dozens of seconds, allowing administration of clonidine 450–600 μg18See insert for the various dosages for pills/vials in Europe vs. the USA. orally (p.o.) with some water19The i.v. route (2 μg.kg-1.h-1 for 24 h) is used if the oral route cannot be used. To our knowledge, the use of oral/nasal/rectal dexmedetomidine in such a setting has not been described. every 4 h.] A similar regimen has been described.[191] [Within 30–120 min, calmness allows restraint to be withheld in young, muscular, combative patients as soon as -1 < RASS < 0 is achieved without any uncoordinated movement or combativeness. As soon as agitation is controlled and tremors are completely suppressed, the regimen is eased to clonidine p.o. 300–600 μg every 6 h, then 9 h and so on (with additional support: Table 1). Usually, a dose of clonidine up to ≈ 2 μg.kg-1.h-1 (i.e., European dosage ≈ 22 vials or pills/24 h/70 kg) allows one to achieve quietness within a few hours.]
  2. **Admission from the ward following benzodiazepine overdose**
    When a DT patient is admitted from the ward to the CCU following oversedation with benzodiazepines, the patient presents a combination of oversedation and/or agitation, a degree of hypoventilation, sepsis (usually pulmonary) and minimal acute kidney injury. Supportive treatment is provided (table 1) and benzodiazepine administration is stopped immediately. Alpha-2 agonists should be administered immediately after physical examination and assessment
    of contra-indications: dexmedetomidine 0.7[200] to 1.5 μg.kg-1.h-1 or clonidine i.v. 1-2 μg.kg-1.h-1 adjusted to –2 < RASS < 0. The low dose (0.7 μg.kg-1.h-1) is used[200] after an unsuccessful haloperidol trial (maximum daily dose of haloperidol: 30 mg (200)). [In our hands, high-dose alpha-2 agonists represent one strategy to control agitation as quickly as possible in the context of pre-existing benzodiazepine or haloperidol treatment (part I, III, F). The alpha-2 agonist is tapered as above (part I, III, F).]
    [Following benzodiazepine overdose and failure to control DT, the objective is to *suppress* agitation, hallucinations, and tremors for ≥ 24 h. This may lead to overshooting when agitation is controlled as quickly as possible, without intubation (transient deeper sedation: -3 < RASS < -2; ≈ 3 h).] Immediately after achieving this deep short-term sedation with an alpha-2 agonist, light sedation (-2 ≤ RASS ≤ 0) is aimed for with lowering the alpha-2 agonist dose: agitation, jerking, and tremors should be totally suppressed in the absence of physical restraint. Non-invasive or invasive ventilation may be considered if required.
- **Established delirium tremens**
  A patient admitted to the CCU presenting with established DT often exhibits multiple trauma and/or organ failure (MOF): pneumonia or ‘focal’ ARDS, oliguria or acute kidney injury, agitation occurring during weaning from conventional sedation and so on. The risk of sepsis or septic shock in a patient with an initial diagnosis of DT persists during or immediately after complete recovery of delirium, with possible delayed diagnostic.
  [In the setting of established DT: a) conventional sedation is stopped immediately before initiation of a dexmedetomidine/ clonidine infusion, b) only i.v. alpha-2 agonists should be used, that is, dexmedetomidine 1.5 μg.kg-1.h-1 adjusted to heavy sedation (–3 < RASS < -2), for the shortest possible interval and lightened as soon as possible. As agitation may persist for 30–180 min before achieving steady sedation with dexmedetomidine, iterative bolus rescue sedation (midazolam)[74] may be administered (part I, III,D).] Conversely, would breakthrough occurs, haloperidol bolus 5-10 mg is considered.
- **Refractory delirium tremens**
  Alpha-2 agonists were used early in the setting of refractory DT.[236, 237, 238] [In this setting, there is no upper limit for the dose of an alpha-2 agonist. The issue is achieving quietness without brisk movements, hallucinations or tremors for ≥ 24
  h. If alpha-2 agonists used alone cannot achieve quietness (dexmedetomidine 1.5 μg.kg-1.h-1 or clonidine 2 μg.kg-1.h-1), then neuroleptics should be added (Table 2)]. High-dose alpha-2 agonists and neuroleptic agents should be adjusted downward as soon as suppression of tremor and agitation ^3^>24 h are observed. [Neuroleptics are selected according to primary symptom (Pichot, unpublished data):
  - hallucinations: haloperidol20Olanzapine vs. haloperidol treatments show no diference. However, an open-label study shows the superiority of dexmedetomidine vs. haloperidol (quoted from (234)). Quietapine, an atypical neuroleptic with a minimal efect on the QT interval, at 50– 200 mg orally or via n/g tube every 12 h appears to be more efective than ‘as needed’ haloperidol to address critical care delirium.[239] 50 mg/48 ml, continuous infusion: 2 mL.h-1 initially, that is, 50 mg.day-1.
  - agitation: loxapine 100 mg through an n/g tube every 6 h to achieve deep sedation (3 < RASS < -2) for 3–6 h and control agitation, followed by adjustment to -2 < RASS < 0 as early as possible (part I, III). A period (≈ 3–6 h) of deep sedation (-3 < RAAS < -2) is usually inescapable, followed by lightened sedation (-2 < RASS < 0). Loxapine 100 mg every 6 h is modified to loxapine every 12 h with the addition of, for example, dexmedetomidine 1.5 μg.kg-1.h-1. Then, the loxapine dosage is reduced as early as possible (part I, III, D).
  In our hands, a combination of high-dose alpha-2 agonists and high-dose neuroleptics abates refractory DT. Baclofen 50–150 mg[144] or low-dose midazolam (e.g., 1 mg.h-1) are never necessary to achieve quietness. As these drugs are GABA agonists and as GABA is involved in alcoholism, this possibility presents nevertheless relevance.]
- Withdrawal from illegal drugs
  Administration of alpha-2 agonists in the setting of heroin withdrawal[240] lead to the use of alpha-2 agonists in the setting of anaesthesia,[45,241,242] alcohol[236] or tobacco withdrawal. [243] Alpha-2 agonists are widely used in rapid detoxification management. Accordingly, early rapid detoxification in methadone addicts has been observed: clonidine (17 μg.kg-1. day-1, i.e., ≈ 1.2 mg.day-1), without general anaesthesia, achieved a high success rate and near-total suppression of withdrawal symptoms.[244]
  For tobacco withdrawal, as most DT patients present mixed alcohol and tobacco intoxication, alpha-2 agonists are administered to achieve the desired sedative efect in addition to transdermal nicotine.

### II) Postoperative sedation

Medical patients require higher doses of alpha-2 agonists. [12] By contrast, postoperative[12] or trauma patients require higher doses of analgesics.[12] These data require replication and emphasize iterative objective evaluations of pain in intubated vs. nonintubated patients.

- **Multiple trauma**
  Multiple-trauma patients (Table 3) experience problems beyond the scope of this manuscript, as: a) definitive surgery and haemostasis; b) inflammation, infection and so on; and c) alcohol, cannabis, heroin and so on withdrawal (part II, I), regardless of comorbidities.
  Pain should be iteratively evaluated by combining behavioural[129] and circulatory parameters. A complete shift of emphasis has emerged: opioid analgesics are currently used only as rescue analgesics,[151] which may evoke exaggerated bradycardia in the presence of dexmedetomidine.[197]
  The analgesics with the fewest side-effects should be selected considering the importance of spontaneous breathing. [Accordingly, opioid-free analgo-sedation should be used: a) intact respiratory genesis evoked by alpha-2 agonists[41,80,88] and b) analgesia[76] and analgognosia[10] evoked by alpha-2 agonists. This lowers the analgesic requirements. Nonopioid analgesics (or opioid analgesics with minimal respiratory effects, such as tramadol) should be considered, that is, ketamine, nefopam, aminotryptilline, pregabalin/gabapentin, carbamazepine and nonsteroidal anti-inflammatory agents (Table 3). Relative contra-indications exist according to age (nefopam), acute kidney injury (tramadol), anti-inflammatory agents, seizures, etc.]
- **Cardiac surgery**
  The standardization of cardiac surgery with/without cardiopulmonary bypass (CPB) led to the establishment of fast-track cardiac anaesthesia and early discharge from the CCU. Patients’ conditions are complex because of extensive comorbidities beyond the scope of this manuscript: age, left ventricular systolic or diastolic impairment, chronic obstructive lung disease, chronic kidney insufficiency, diabetes, and obesity and so on (Table 4).

**Table 3:** Multiple trauma

Overall assessment	Iterative use of Richmond Agitation Sedation scale and Behavioural Pain Scale.
	Circulation: iterative peripheral mottling, capillary refill, diuresis, passive leg raising.
	Iterative echocardiography: normalize volemia before administration of alpha-2 agonists.
	Ventilation: assess ‚wet’ lung/ARDS following multiple transfusion.
	NB: ‚wet’ lung or peripheral edema does not contra-indicate the use of alpha-2 agonists; alpha-2 agonists evoke anti-ADH effect, diuresis and improved kidney function.
	Kidney, liver, metabolic function.
	Infection, inflammation.
Supportive treatment	Ventilation: as appropriate, O2 supplementation or High Oxygen flow (Optiflow®) or non-invasive ventilation or invasive ventilation as soon as sedation is achieved.
	Hydration to adequate diuresis (e.g. > 1 mL.kg.d, i.e., > 1700 mL/70 kg/24 h) vs. renal replacement therapy, if appropriate.
	After full haemostasis, prophylaxis of thrombosis.
	Prophylaxis of gastro-intestinal haemorrhage.
	Supportive treatment specific to considered trauma.
	Consider BIS or equivalent if benzodiazepine or propofol infusion are to be used.
Sedation	1) Goal : quietness (intubated patient: -3 < RASS < 0; non-intubated patient: -2 < RASS < 0), analgesia (BPS < 3–5), spontaneous ventilation (e.g., pressure support with minimized work of breathing: pH, PaCO2) as soon as possible.
	1) Discontinue propofol, benzodiazepine, opioid analgesics.
	2) Dexmedetomidine 0.75 μg.kg-1.h-1 up to 1.5 μg.kg-1.h-1 adjusted to -3 < RASS < 0.
	NB: if sedation is not sufficient with the alpha-2 agonist, do not EVER administer a bolus of alpha-2 agonist : use ‚rescue’ sedation to be repeated if necessary and increase the administration of i.v. continuous dexmedetomodine up to ‚ceiling’ effect (1.5 μg.kg-1.h-1).
	Insert a sticker ‚DO NOT BOLUS’ on the i.v. line (Shehabi 2010).
	3) If insufficient, loxapine 100 mg through n/g adjusted to, for example, 25 mg*4 or haloperidol 1 mg.h-1 lowered to 0.25-0.5 mg.h-1 adjusted to -1 < RASS < 0.
	4) Non-opioid analgesia:
	Ketamine 50–100 mg.day-1, tramadol 400 mg.day-1, nefopam 100 mg.day-1/48 ml : 2 mL.h-1. These dosages may be reduced to 1 mL.h-1 then 0.5 ml.h-1 after 1-3 days of administration. The clinical impression is that after full impregnation with an alpha-2 agonist for 1-3 days, the patient needs little analgesia per se, presumably due to the analgognosia evoked by the alpha-2 agonist (see text).
	NB: In elderly patients administer nefopam 20mg/day for 1–2 days then increase nefopam if necessary up to 100 mg if no cognitive side-effects occur. Beware of possible acute urine retention if Folley catheterization is not performed.
	NB: Tramadol is a weak opioid analgesics acting on μ receptors, contra-indicated if acute kidney insufficiency is present
	To avoid completely opioid analgesics or for an early stop of the administration of tramadol-nefopam especially when elderly patients are considered, consider:
	amitryptyline (Laroxyl®) 25 mg i.v.*4 or lidocaine 0.5 mg/kg/h (loading dose: 1 mg. kg-1.h-1) or ketamine (0.25 mg kg-1.h-1) infusion.
	or pregabaline (Lyrica®) 150–600 mg/day : start with 25 mg*2 through n/g (day 0), then 50*2 (day 2) then 75*2 (day 4). When pancreatitis or CCU neuromyopathy is considered: 150*2 and up to total daily dose: 600 mg.
	or gabapentine (Neurontin® 100–900 mg/day) or carbamazepine (Tegretol® 200–400 mg/day).
	5) ‚Rescue opioids’: if needed, opioid analgesics to be re-introduced sparingly; allow for early spontaneous ventilation, absence of effect on intestinal motility, hyper-algesia.
Rescue sedation	To avoid making a complex situation more complex , conventional sedation is to be discontinued abruptly. In intubated mechanically ventilated patients, as i.v. dexmedetomidine or clonidine evoke sedation after ≈ 60 to 180 min respectively, ‚rescue’ sedation (midazolam bolus 3–5 mg) is to be administered repeatedly as required until the alpha-2 agonist evokes quietness to -1 < RASS < 0, combined with a neuroleptics, if needed.
	Immediately prior to nursing, ‚rescue’ sedation (midazolam 1–2 mg (titrated to effect) may be administered to maintain -3 < RASS < 0.

**Table 4:** Postoperative management following cardiac surgery

Positive diagnosis	Functional history, preoperative catheterization/coronary angiography or echocardiography, intra-operative echocardiography findings, surgical procedure, cross-clamp and cardiopulmonary bypass time, administration of cardioplegia (volume, interval), vasopressors, inotropes, antiarrhythmics, blood losses and products.
Overall assessment	Circulation: iterative assessment of bleeding, mottling, capillary refill, diuresis, ECG, troponin, echocardiography and blood gases (arterial and central venous); iterative assessment of A-V block, volemia, compliance, contractility.
	Ventilation: chest X-ray or lung echography.
	Kidney/metabolic function.
	Consider BIS or equivalent if benzodiazepine or propofol infusion is to be used, especially if low cardiac output occurs.
Supportive treatment	Address ventilation/chest X ray, volume, inotropic and vasopressor/dilator status.
	Peripheral external rewarming.
	Suppress shivering (bolus of i.v. meperidine 100 mg or clonidine 37.5 μg i.v.).
Sedation	1) Goal: extubation as soon as possible in a quiet unpainful patient: -1 < RASS < 0 after addressing normothermia, bleeding, circulation, ventilation.
	2) Discontinue anaesthesia.
	3) Dexmedetomidine 0.75 μg.kg.h-1 adjusted to -1 < RASS < 0 (contra-indications: A-V block II-III unless pacing in place, hypovolemia).
	Hypotension : check inotropism and volemia, then low dose noradrenaline 0.001.005 μg.kg.min-1, if critical stenosis exists.
	NB : Alpha-2 agonists have repeatedly been shown to increase sensitivity to noradrenaline and dobutamine in the setting of cardiac surgery.
	4) Extubation as early as possible, ideally within OR, following end of rewarming and absence of bleeding.
	5) Discontinue alpha-2 agonist as soon as possible (usually next morning): absence of tachycardia or hypertension, spontaneous ventilation.
	6) Analgesia :
	Intraoperatively and before emergence: paracetamol 1g, nefopam 20, ketoprofen 50 mg depending upon high dose opioid anaesthesia vs. opioid free anaesthesia.
	Consider non-opioid analgesics: ketamine 50 mg.day-1, tramadol 400 mg.day-1, nefopam 100 mg.day-1 for 48 h.
	To completely avoid opioid analgesics or to achieve early stop of the administration of tramadol-nefopam in elderly patients, consider amitriptyline (Laroxyl® 25 mg*4), gabapentine (Neurotin® 100-900 mg.day-1), pregabaline (Lyrica® 150-600 mg.day-1), carbamazepine (Tegretol 200-400 mg.day-1).
	7) If not sufficient, use morphine (‚rescue opiates’) sparingly following administration of non-opioid analgesics.
	NB: If sedation is not sufficient with the alpha-2 agonist, do not EVER administer a bolus of alpha-2 agonist : use ‚rescue’ sedation (midazolam 3–5 mg) to be repeated if necessary and increase the administration of i.v. continuous dexmedetomodine up to „ceiling” effect (1.5 μg.kg-1.h-1).
Rescue sedation	Upon CCU admission, as dexmedetomidine requires ≈60 min to evoke sedation, if needed, midazolam bolus 3–5 mg or propofol 10-20 mg, repeated as required, to achieve -1 < RASS < 0: the patient should stay quiet to allow for transfer, nursing and assessment. Consider extubation as soon as temperature, circulatory and ventilatory stability is achieved.
	NB: Use of propofol bolus as opposed to midazolam bolus in the presence of alpha-2 agonist exposes to a higher risk of hypotension and bradycardia

[Cooperative sedation with an alpha-2 agonist will allow discontinuation of opioid analgesia intraoperatively or upon reaching the CCU with appropriate use of rescue sedation. In addition, opioid-free anaesthesia is now used in the cardiac anaesthesia setting.[245] Reduced cognitive and respiratory effects due to minimal anaesthesia or opioid-free anaesthesia will ease intraoperative and postoperative circulatory instability. Cooperative sedation with opioid-free postoperative analgesia (Table 4) will achieve circulatory stability with adequate analgesia, analgognosia and sympathetic deactivation evoked by alpha-2 agonists. The alpha-2 agonist is preferably administered as a premedication.[45,184] Conversely, the alpha-2 agonist can be administered either intraoperatively before or upon admission to the CCU.[184,246]

## Conclusion

Alpha-2 agonists are to be used as first-line sedatives, based on individual assessment and dosage, not on a ‘one size fits all’ approach. By easing cognitive, ventilatory and circulatory management, a wise use of cooperative sedation will ease the overall CCU stay.

### Insert I: Functional anatomy of alpha-2 adrenergic receptors

*Neurochemistry*:

Alpha-2 agonists act on alpha-2 adrenergic receptors located on or near the central noradrenergic and peripheral sympathetic neurons: a) ‘presynaptic’ *autoreceptors* on central noradrenergic/adrenergic cell bodies or terminals and on sympathetic post-ganglionic neurons in the peripheral sympathetic system; b) ‘post-synaptic’ *heteroreceptors* located on *non*-noradrenergic or *non*-adrenergic neurons or on neurons acting peripherally on post-ganglionic sympathetic neurons (Figure 3).21The matter is complex. Suppression of central noradrenergic/ adrenergic cell bodies (i.e., of autoreceptors) do not suppress the cognitive[216] or antihypertensive[50] effects of alpha-2 agonists. Therefore, the effects of alpha-2 agonists are consecutive to actions on non-adrenergic neurons. They presumably act on multiple cell types in the brain stem,[219] that is, on auto- and heteroreceptors on noradrenergic and non-adrenergic neurons respectively.

*Anatomy*:

In the CNS, alpha-2 agonists act:

- a) on the ventral noradrenergic bundle, which projects from the rostral ventrolateral medulla (glutamatergic and adrenergic C1 neurons within the ‘vasomotor centre’: pre-sympathetic neurons) to the spinal intermediolateral cell column, where sympathetic pre-ganglionic neurons originate from (Figure 1). The alpha-2 agonist evokes sympatho-inhibition in the healthy resting supine volunteer. In the CCU patient, sympathetic deactivation occurs which normalizes the sympathetic activity back toward baseline.
- on the dorsal noradrenergic bundle, which projects from major noradrenergic nuclei (locus coeruleus-A6, etc.) to rostral sites (cortex, hippocampus, etc.) (Figure 1). This explains the sedative and antipsychotic effects of alpha-2 agonists to counteract the ‘CCU triade’: pain, altered cognition/delirium, agitation[234] (Figure 2).
- on or near cardiac parasympathetic neurons (cholinergic ‘cardiac vagal motoneurons’: CVM located next the vasomotor centre: nucleus ambiguous ventro-lateral[247]), which projects on the sinus node). The alpha-2 agonist generates cardiac parasympathomimetic effects[145] (cardiac parasympathetic activation: bradycardia; Figure 3).

**Figure 4:**
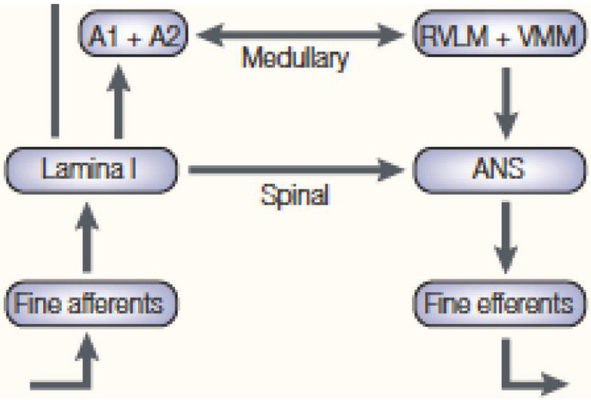
**Hierarchical organization of neural homeostasis involving the sympathetic nervous system at lower brain stem and spinal levels only**: Small-diameter afferent fibres that report the physiological conditions of all of the tissues of the body terminate in lamina I of the spinal dorsal horns. The ascending projections of lamina I neurons provide the bases for somato-autonomic reflex arcs at the spinal and medullary levels (e.g., ‘somato-sympathetic reflex’ evoking hypertension and tachycardia following a nociceptive stimulus). By contrast, at the spinal levels, lamina I projects strongly to the sympathetic regions in the intermediolateral cell columns of the thoracolumbar cord, where the sympathetic preganglionic neurons originate (ANS: autonomic nervous system; direct spinal somato-sympathetic reflex). In the medulla, lamina I neurons project to the A1 and A2 catecholaminergic cell groups (RVLM: rostral ventrolateral medulla, i.e., vasomotor centre; VMM: ventromedial medulla). Modified from Craig, Nature Neuroscience, 2002, 3, 655–66.[231]

*Physiology*:

Central noradrenergic and adrenergic systems are included within three hierarchized systems (Figures 1 and 4) coordinating the behavioural and autonomic adjustments to the external environment:

- **‘autonomic’ loop**: the ascending pain pathways branch in the lower brain stem on the vasomotor centre,[248] which in turn projects to the spinal intermediolateral cell column; this explains the lowered cardiac parasympathetic activity (cardiac parasympathetic withdrawal) and increased sympathetic activity occurring during a nociceptive stimulus (somato-sympathetic reflex[249,250]) (Figure 1).
- **‘pain’ loop[231]**: the ascending pain pathways branch in the upper brain stem on the descending noradrenergic and serotoninergic pathways. These descending noradrenergic and serotoninergic pathways in turn suppress ascending pain messages within the spinal lamina I-II (‘gate control’ theory; Figure 4). Therefore, alpha-2 agonists coordinate pain suppression and autonomic effects (e.g., sympathetic de-activation or parasympathetic activation) simultaneously via *different* loops.
- the rostral projections originating from the locus coeruleus to the dorsal noradrenergic bundle and cognitive brain helpexplain the anti-delirium properties,[225,226,251] possibly a direct effect of alpha-2 agonists (Figure 2).

### Insert II: Addressing hypovolemia before administration of alpha-2 agonists

**Figure 5:**
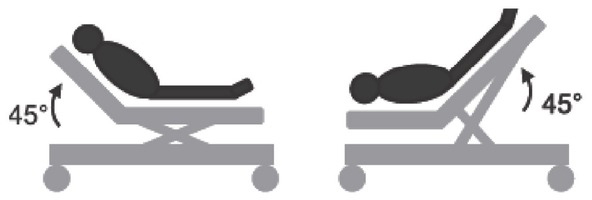
**Passive leg raising** (PLR) while pivoting the entire bed causes no hip flexion (no sympathetic activation) and evokes a larger transfer of blood from the unstressed venous blood volume to the heart. Changes in cardiac output evoked by PLR predict the response of cardiac output to volume expansion in the setting of circulatory failure. The specificity of PLR is acceptable when changes in pulse pressure are assessed; however, the sensitivity is poor.[233] Modified from Monnet, Intens Care Med, 2008, 34: 659–63.[170]

Two different issues are to be considered:

1. how to assess and address hypovolemia when noradrenaline is used and sympatholytics superimposed?
  This is simple, nevertheless *meticulous*.
  Within 30–90 s after PLR, systemic blood pressure, vena cava,22Ventilatory-evoked changes of the diameter of the vena cava: End-expiratory inferior vena cava diameter IVCEE < 8 mm predicts fluid responsiveness with a 95% specificity. Conversely, IVCEE > 28 mm predicts absence of fluid responsiveness with 95% specificity.[171] and if possible, aortic velocity are observed. PLR is used as a ‘reversible volume load’ and performed so as to minimize the changes in pain-evoked sympathetic activity (hip flexion), and heart rate and its consequences on the venous capacitance[170] (Figure 5). Then the alpha-2 agonist is administered: 0.125 μg.kg-1.h-1 then 0.25 and so on, up to 1.5 μg.kg-1.h-1 over 6–12 h (part I, III, F). Systemic pressure, vena cava and aortic velocity responses to PLR are addressed iteratively before each increment of alpha-2 agonist. As the patient is going to stay in the CCU for an extended stay, with rescue sedation at hand, a steady state cooperative sedation is not required upfront, but circulatory stability the goal. Our approach is overly cautious, bearing in mind preload-dependent vs. pre-load independent patients (see below). Nevertheless, more liberal prescription regimen[40] fits with the requirement to maintain the venous return, so that the *stroke volume is maintained and exaggerated bradycardia avoided*, especially when positive pressure ventilation with positive end-expiratory pressure is set up.
2. what is the physiological and pharmacological rationale behind such management? The matter is complex, involving capacitance vs. resistance vessels, alpha-1 vs. alpha-2 receptors, pre-load dependence, sympathetic block vs. normalization and the considered disease (cardiogenic vs. septic shock).

**Figure 6:**
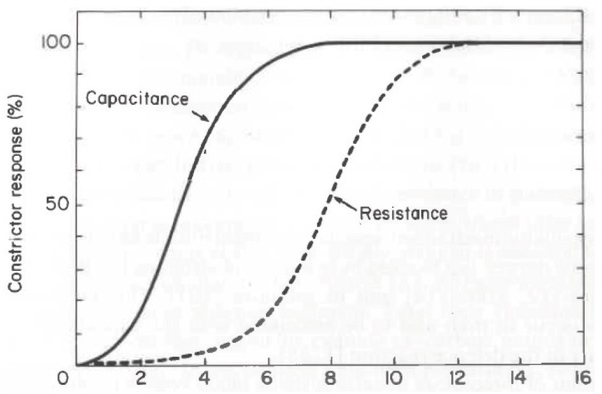
**Frequency-response curve deduced for resistance (dashed) and capacitance (continuous) in cat skin-muscle**. Effects of lumbar vasoconstrictor fibre stimulation calculated in a percentage of the maximum response for the 2 vascular sections. The curve for the capacitance vessels (veins) is to the left: this curve implies more pronounced effects in this section in the low frequency range, compared with those in the resistance vessels (arteries). *One third of the regional blood volume is expelled at a low frequency stimulation rate, thus increasing venous return*. A fully developed response occurs within 30–40 s for both resistance and capacitance vessels. Immediate relaxation occurs after cessation of sympathetic stimulation. In contrast, there is delayed relaxation at high rates of sympathetic stimulation; this delayed relaxation could be of relevance after prolonged administration of high-dose noradrenaline, for example, in septic shock: prolonged vasomotor sympathetic hyperactivity is associated temporally with poor microcirculation. In turn, prolonged impaired microcirculation is associated with increased mortality.[139] Would normalization of vasomotor sympathetic activity back toward baseline levels be observed before shock, and would improve microcirculation improve outcomes? Modified from Mellander, Acta Physiol Scand, Suppl, 1960, 50: 1–86[61] and Prys-Roberts, Regulation of Circulation, In The circulation in anaesthesia: applied *physiology* and pharmacology, Blackwell, Oxford, 1980, pp 179–207.[232]

*Physiology*: Capacitance vessels (veins) reach 90% of maximum vasoconstriction at *low* rates of sympathetic stimulation[61] (Figure 6). By contrast, for a similar low rate of sympathetic stimulation, the resistance vessels (arteries) are only 10% constricted[61] (Figure 6). Thus, minimal changes in low levels of sympathetic activity evoke proportionally more changes in the veins than in the arteries. In fact, low frequency sympathetic discharge evokes large relative increase in HR, and positive inotropic effect. This generates a cardiovascular *coordination* in the healthy supine volunteer occurring, firstly, at *low* frequency sympathetic discharge.[61] During upright positioning (‘head-up tilt’), increased venous return, HR and contractility occur. Higher frequency of sympathetic discharge reinforces this sequence of events. By contrast, the cardiac parasympathetic system is: a) deactivated during head-up tilt, leading to tachycardia within 1–2 s,[230] and b) activated briskly during cessation of exercise[252] with exaggerated bradycardia.

Pharmacology: In the setting of septic shock, systemic administration of noradrenaline squeezes the venous capacitance and increases venous return.[175] Contractile response of vascular smooth muscle to NA may be initiated on the arterial side by alpha-1 receptors and on the venous side by alpha-2 receptors.[253] Accordingly, *in vitro* administration of an alpha-2 agonist leads to veno-constriction.[254] Conversely, normalization of vasomotor sympathetic hyperactivity towards baseline, with alpha-2 agonists, reduces the release of endogenous NA in the synaptic cleft, increases the venous capacitance and decreases the venous return. The physiological consequences are unclear: does normalized sympathetic activity lead to passive venous dilation via reduced NA agonism on alpha-2 receptors? Or to passive arterial dilatation via reduced NA agonism at the level of alpha-1 adrenergic receptors? Or both? The clinical consequences are clear: alpha-2 agonists in the hypovolemic patient reduce the venous return, leading potentially to catastrophes, unless volemia is iteratively checked.

When preload-dependent, the patient moves from the flat part of the Frank-Starling curve to the steep part, implying reduced central venous pressure and lowered cardiac output.

- Propofol 1 mg.kg-1.h-1 generates pre-load dependence: 14 patients out of 22 preload-independent patients became pre-load dependent, after propofol infusion.[255]
- Dexmedetomidine 0.7 μg.kg-1.h-1 does not generate as much pre-load dependence: only 5 patients out of 20 preload-independent patients became preload dependent.[255] Accordingly, no side effects were observed when dexmedetomidine 0.7 μg.kg-1.h-1 was administered in patients presenting with septic shock (NA requirements ≈ 0.69 μg.kg-1.min-1, i.e., ≈ 3 mg.h-1/70 kg), *with additional volume to maintain stroke volume* in 7 out of 38 patients.[40]

*Clinical setting*: The swiftness and amplitude of autonomic response to brisk behavioural or environmental demands is lost in aged or CCU patients. Furthermore, Anaesthetics and conventional sedation elicit sympathetic block (as with regional anaesthesia), further suppressing this coordination. By contrast to anaesthetics, alpha-2 agonists shift the response-curve to higher frequency without blocking the noradrenaline release: stronger stimuli (hypovolemia, haemorrhage) overcome centrally this shift and the sympathetic normalization evoked by alpha-2 agonists. Higher levels of sympathetic activity elicit venous or arterial constriction. To sum up, under alpha-2 agonists, the peripheral alpha- and beta-adrenergic receptors are left unblocked. By contrast, beta-blockers block peripheral beta receptors.

When volume loading is not administered before general anaesthesia and intubation,[169] the venous capacitance is not replenished *before* sympathetic deactivation: cardiac filling pressure is lowered and stroke volume reduction occurs. As alpha-2 agonists reduce right and left[104,105] filling pressures, but increase left compliance[103] and systolic [109] performance, they evoke

- detrimental side-effects in the setting of hypovolemia (trauma, sepsis): superimposed on hypovolemia, sympathetic deactivation reduces the venous return and evokes pump failure. Lowered return and lowered impedance may increase dynamic outflow obstruction.
- beneficial side-effects in the setting of cardiogenic pulmonary oedema: lowered filling pressure, improved diastolic and systolic performance.

## Abbreviations and glossary

APRV: airway pressure release ventilation
A-V block: atrio-ventricular block
ARDS: acute respiratory distress syndrome
BIPAP: bilevel positive airway pressure
BIS: bispectral index, computerized electroencephalogram bpm: beats per min
CAM-ICU: confusion assessment method for intensive care unit Cardiac parasympathetic activity: ‘cardiac vagal activity’, activity of the vagus projecting to the heart
Cardiac: sympathetic activity: activity of the sympathetic nervous system projecting to the heart
CCU: critical care unit
CMV: controlled mechanical ventilation
CVM: cardiac vagal motoneurons, that is, cardiac parasympathetic motoneurons located in the lower brain stem (nucleus ambiguous) and projecting to the sinus node Cooperative sedation: sedation evoked by alpha-2 agonists
CT: computerized tomography
DT: delirium tremens
HR: heart rate
LC: locus coeruleus, noradrenergic A6 group located in the upper brain stem projecting to rostral brain and dorsal horn
NIV: non-invasive ventilation
P/F: PaO_2_/FiO_2_
PLR: passive leg raising
PS: pressure support
MAP: mean arterial pressure
n/g: nasogastric
NMB: neuromuscular blocker
RASS: Richmond Agitation Sedation Scale
RRT: renal replacement therapy
RVLM: rostral ventrolateral medulla, ‘vasomotor centre’, projecting to the sympathetic preganglionic neurons in the intermediolateral cell column.
SsvcO2: O2 saturation in the superior vena cava
TBI: traumatic brain injury
TEE: transoesophageal echocardiography
TIVA: ‘light’ total intravenous anaesthesia, analgo-sedation, conventional sedation
TTE: transthoracic echocardiography Vasomotor sympathetic activity: activity of the sympathetic system directed toward veins and arteries.
VAS: visual analogue scale
VO2: O_2_ consumption
